# *Cyclin G* Functions as a Positive Regulator of Growth and Metabolism in *Drosophila*

**DOI:** 10.1371/journal.pgen.1005440

**Published:** 2015-08-14

**Authors:** Patrick Fischer, Martina K. La Rosa, Adriana Schulz, Anette Preiss, Anja C. Nagel

**Affiliations:** Institute of Genetics, University of Hohenheim, Stuttgart, Germany; The University of North Carolina at Chapel Hill, UNITED STATES

## Abstract

In multicellular organisms, growth and proliferation is adjusted to nutritional conditions by a complex signaling network. The Insulin receptor/target of rapamycin (InR/TOR) signaling cascade plays a pivotal role in nutrient dependent growth regulation in *Drosophila* and mammals alike. Here we identify Cyclin G (CycG) as a regulator of growth and metabolism in *Drosophila*. *CycG* mutants have a reduced body size and weight and show signs of starvation accompanied by a disturbed fat metabolism. InR/TOR signaling activity is impaired in *cycG* mutants, combined with a reduced phosphorylation status of the kinase Akt1 and the downstream factors S6-kinase and eukaryotic translation initiation factor 4E binding protein (4E-BP). Moreover, the expression and accumulation of *Drosophila* insulin like peptides (dILPs) is disturbed in *cycG* mutant brains. Using a reporter assay, we show that the activity of one of the first effectors of InR signaling, Phosphoinositide 3-kinase (PI3K92E), is unaffected in *cycG* mutants. However, the metabolic defects and weight loss in *cycG* mutants were rescued by overexpression of Akt1 specifically in the fat body and by mutants in *widerborst* (*wdb*), the B'-subunit of the phosphatase PP2A, known to downregulate Akt1 by dephosphorylation. Together, our data suggest that CycG acts at the level of Akt1 to regulate growth and metabolism via PP2A in *Drosophila*.

## Author Summary

Size and growth of an organism are adjusted to nutritional conditions by a complex regulatory network involving the Insulin receptor and TOR signaling cascades. *Drosophila melanogaster* has been used in the past as a genetically tractable model to unravel the complex circuitry by genetic means. We have identified CycG as an important player in the regulation of TOR signaling. CycG mutants are underweight in the midst of food and show typical signs of TOR defects. We provide evidence that CycG acts at the level of Akt1 kinase that links the Insulin receptor and TOR signaling cascades. Molecular and genetic data point to an interplay of CycG and phosphatase PP2A, a well established negative regulator of Akt1 activity. Moreover, CycG may influence PP2A-Akt1 binding. We propose that CycG, by impeding PP2A-Akt1 interaction, acts as a positive regulator of growth in *Drosophila*.

## Introduction

The growth of an organism is a highly coordinated process regulated by a wide range of different inputs. Members of the Insulin receptor (InR) and Target of rapamycin (TOR) signaling pathways are well established key players in the control of cell growth in higher eumetazoa. Studies in different organisms support the idea that this signaling network modulates cellular growth in response to nutrient availability, growth factor signaling, energy status as well as to diverse cellular stressors (for review [1], [2]). *Drosophila* has proven to be a powerful system for investigating the InR/TOR signaling network. Signaling through the InR pathway is triggered through the binding of *Drosophila* Insulin-like peptides (dILPs) to the single InR. Four of the eight known dILPs (dILP1, 2, 3 and 5) are expressed in neurosecretory cells of the brain, the so-called Insulin producing cells (IPCs), from which they are released to act systemically via haemolymph transport [3]-[7]. Activation of the InR triggers a phosphorylation cascade mediated by a relay of kinases. As one of the first steps, the lipid kinase Phosphoinositide 3-kinase (PI3K92E) is activated leading to the activation of the kinase Akt1 that in turn phosphorylates the small GTPase Rheb (Ras homologue enriched in brain), an activator of TOR (for review [2], [8]). The TSC1/2 (tuberous sclerosis complex) tumor suppressor complex inhibits the activity of the TOR kinase by negatively regulating Rheb [9]-[13]. In addition, phosphatases like PTEN and PP2A were identified as negative regulators of the InR/TOR signaling cascade [14]-[18].

Starvation, especially amino acid withdrawal, interferes with dILP secretion in larval brains: dILPs accumulate in IPCs, and larval growth is impaired [4]. Nutrient availability is sensed by the TOR network which serves as the central coordinator of cellular and organismal growth, aging and fertility [19]-[21]. The TOR kinase, central to the TOR pathway, exists in two distinct conserved complexes, TORC1 and TORC2. Like in mammals, TORC1 is the crucial regulator of cell size and organismal growth in *Drosophila* (for review [2], [22]). The best studied substrates for TORC1 are S6 kinase (S6K) and the eukaryotic translation initiation factor 4E binding protein (4E-BP), both serving the regulation of translation (for review [22]). Phosphorylation of either protein enhances translation efficiency, either by relief of translational repression as in the case of 4E-BP, or by enhancement of ribosome recruitment as in the case of S6K (for review [1], [23]). This spectrum of phenotypes conforms to the pivotal role of TOR signaling in the control of growth and maintenance of cellular homeostasis in synchrony with the actual nutrient conditions. In *Drosophila* the larval fat body, a functional equivalent of the vertebrate liver and white adipose tissue, acts as a nutrient sensor controlling dILP release in the brain [4], [21]. In accordance, reducing TOR signaling specifically in the fat body has a negative impact on the overall growth of the animal comparable to the effects observed in underfed larvae [4].

Here we identify Cyclin G (CycG) as a new regulator of InR/TOR signaling activity in *Drosophila*. Homozygous *cycG* mutant flies are viable, however females are sterile. Mutant eggs display dorso-ventral patterning defects in the eggshell due to an impaired EGFR-signaling activity. This phenotype was shown to be a consequence of compromised double strand break repair, assigning CycG a role in meiotic checkpoint control during oogenesis [24], [25]. Moreover, CycG was proposed to act as a negative regulator of cell growth and cell cycle progression based on the misregulation of CycG activity [26]. Here we report that the phenotypes of *cycG* mutants recapitulate defects in InR/TORC1 signaling. Our genetic and molecular data indicate that CycG acts at the level of Akt1 presumably via a regulation of PP2A-Akt1 binding. Altogether our genetic and molecular observations provide evidence that CycG is required for InR/TORC1 pathway members to tap their full potential in mediating growth and metabolism in *Drosophila*.

## Results

### *cycG* mutants display defects in growth regulation

**Fig 1:**
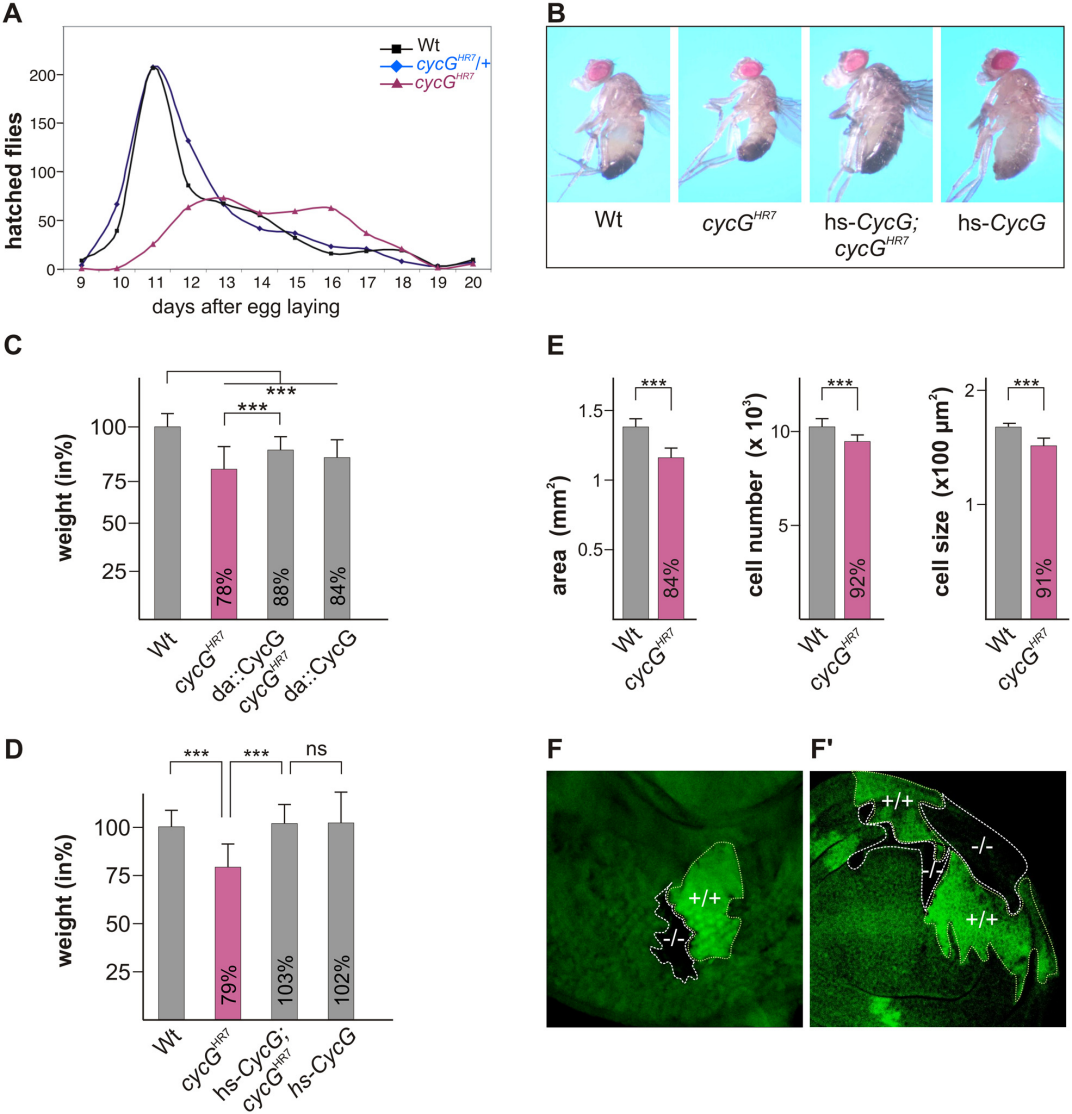
Growth and weight deficits in *cycG* ^*HR7*^ mutant animals. (A) Hatching rates of *cycG* ^*HR7*^ homozygotes (red) versus heterozygous (blue) and homozygous control siblings (black) within 20 days are shown. Results from six parallel crosses were sampled (total n = 2161). Number of heterozygotes was halved to ease comparison. Note developmental delay and reduced survival rates of the *cycG* ^*HR7*^ homozygous mutants. (B) *cycG* ^*HR7*^ mutant animals have a reduced body size which is rescued by hs-*CycG* at ambient temperature. (C) Weight measurements of adult males depicted in relation to wild type animals (Wt) which were set to 100%. Both, loss of CycG (*cycG* ^*HR7*^) and ubiquitous overexpression of CycG (*da*-Gal4::UAS-*CycG*) result in a significant weight reduction. However, in the combination, the weight deficit is significantly ameliorated and not further decreased, reflecting a rescue of CycG loss. (D) The weight of male animals is shown in percent of the wild type. Depicted are wild type (Wt), *cycG* ^*HR7*^ homozygous mutant, hs-*CycG; cycG* ^*HR7*^ and hs-*CycG* flies at ambient temperature. (C-D) Error bars denote standard deviation [n = 100 per genotype]. ***p<0.001; ns: not significant according to Student’s T-test. (E) Compared to the wings of wild type males (Wt, grey bars) wing area, cell number and cell size is reduced in *cycG* ^*HR7*^ mutant males (red bars). Error bars denote standard deviation [n = 12 per genotype]. ***p<0.001 according to Student’s T-test. (F-F') Twin spots of wild type (marked by beta-Galactosidase in green, +/+) and *cycG* ^*HR7*^ mutant cells (marked by the loss of beta-Galactosidase,-/-) were induced in larval eye (F) and wing (F') imaginal discs of heterozygous animals.

Homozygous *cycG* ^*HR7*^ null mutants are viable but female sterile [24]. They are, however, developmentally delayed and underrepresented with regard to their siblings (Fig 1A). In addition, *cycG* ^*HR7*^ mutant animals are smaller and slimmer than the controls (Fig 1B), and have a reduced body weight (Fig 1C). This finding was unexpected as CycG was reported to function as a negative regulator of growth and proliferation based on overexpression studies [26]. We also observed that the ubiquitous overexpression of *CycG* (*da*::*CycG*) resulted in a weight loss, however, was able to ameliorate the weight deficit of the *cycG* ^*HR7*^ homozygotes (Fig 1C). Because of its ability to bind to several cyclin dependent kinases [26]-[28], a strong *CycG* overexpression is likely to interfere with cell cycle regulation, which may explain these observations. In accordance, the subtle induction of a heat shock *CycG* construct (hs-*CycG*) at ambient temperature was sufficient to robustly rescue the observed growth and weight deficits in *cycG* ^*HR7*^ mutant animals (Fig 1B and 1D).

The size defect of the *cycG* ^*HR7*^ mutant animals was further studied in the wing. Here the reduced size was associated with a reduction in cell size and cell number (quantified via trichome density) (Fig 1E), pointing to a defect in InR/TORC1 signaling [29]. Next we induced mutant clones in the developing imaginal tissue by Flp/FRT mediated mitotic recombination [30]. By 72 hours of larval development, the majority of *cycG* ^*HR7*^ mutant cell clones was smaller than their wild type twin spots in eye-antennal as well as in wing discs (Fig 1F and 1F'), indicating a cell autonomous requirement of CycG for normal growth.

In order to exclude second site defects in the *cycG* ^*HR7*^ allele, a second independent *cycG* allele was generated by ‘ends out’ homologous recombination [31]: in the resultant allele *cycG* ^*eoC*^, nearly all of the coding region is deleted (S1A Fig). Like *cycG* ^*HR7*^, the *cycG* ^*eoC*^ mutant as well as the transheterozygous *cycG* ^*HR7*^/*cycG* ^*eoC*^ combinations behave also as protein null on western blots (S1B Fig) and display the same phenotypic characteristics as *cycG* ^*HR7*^, i.e. female sterility with defective egg patterning, developmental delay and a reduced body size and weight (S1C and S1D' Fig). Taken together, our results support a role for CycG as a positive effector of growth/weight control in the fly.

### The fat metabolism is disturbed in *cycG* mutants

**Fig 2:**
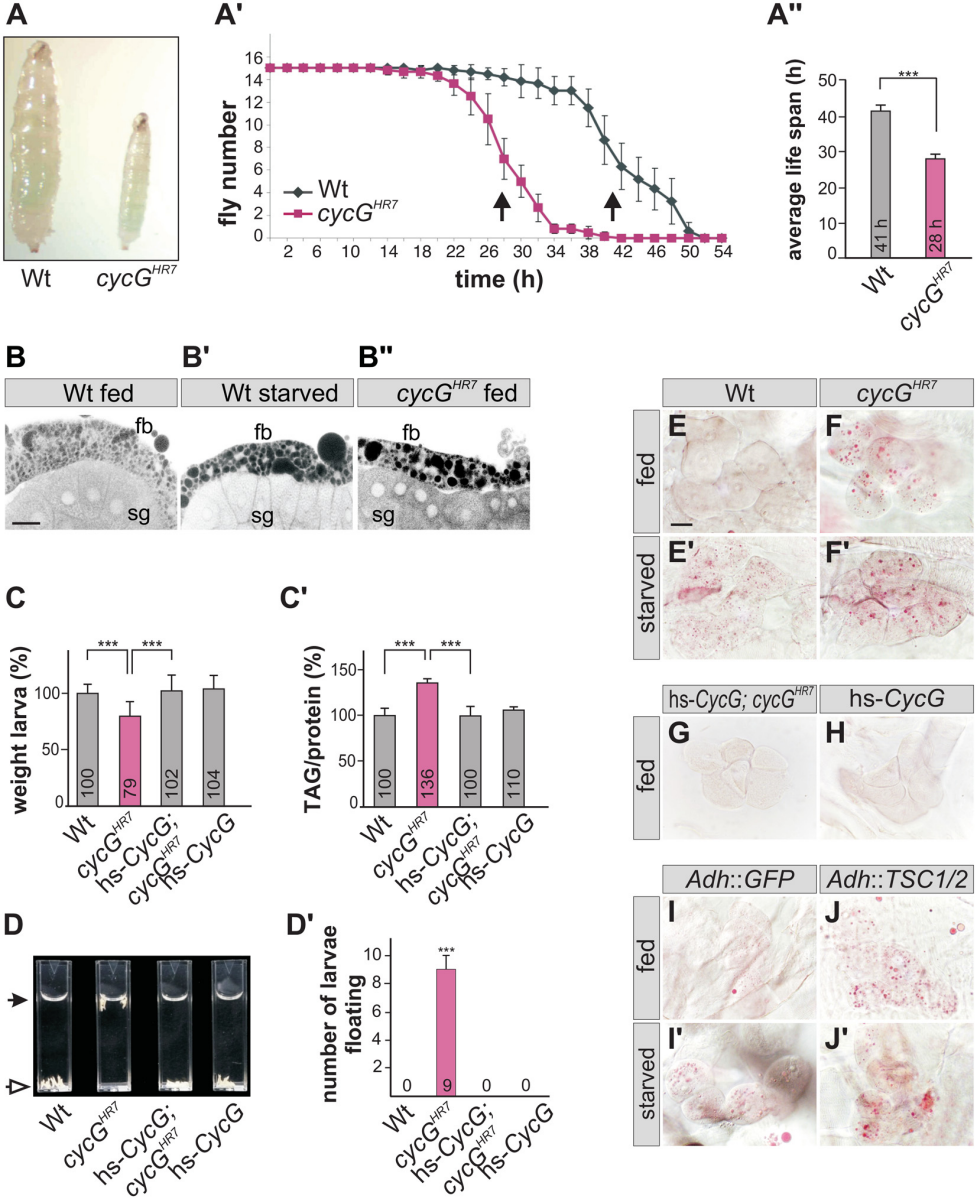
*cycG* ^*HR7*^ mutant animals show defects in fat metabolism. (A) Wild type (Wt) and *cycG* ^*HR7*^ mutants similarly ingest food as visualized by blue colored yeast in the larval gut. (A') Survival rate upon wet starvation was measured in *cycG* ^*HR7*^ mutant males (red) compared to wild type (Wt, black). Sixfold experiment [n = 15]. (A'') The average survival rate for *cycG* ^*HR7*^ is ca 28 hours and for the wild type ca 41 hours, and was taken as the inflexion point of the curve (50% dead animals, arrows in A'). ***p<0.001 according to Student’s T-test. (B-B'') Small lipid droplets are seen in the fat body of a well-fed wild type larva (B, Wt fed). Droplet size increases after of 2 days of amino acid deprivation (B', Wt starved). Similar effects are seen in a well fed *cycG* ^*HR7*^ mutant larva (B'', *cycG* ^*HR7*^ fed). B-B'' represent Nile Red staining of salivary glands (sg) and the adjacent fat body (fb) and were inverted for better visibility. Scale bar: 50 μm. (C) The weight of third instar larvae of the given genotype is depicted in percent of wild type (Wt). Error bars denote standard deviation [n = 100 per genotype]. ***p<0.001 according to Student’s T-test. (C') Histogram depicting TAG content normalized to protein content of the respective larvae shown in C). Wild type levels (Wt) were taken as 100%. Note increase of TAG content in *cycG* ^*HR7*^ mutant larvae by 36% compared to the wild type. Error bars denote standard deviation [n≥3 experiments per genotype]. ***p<0.001 according to Student’s T-test. (D) In contrast to the wild type (Wt, open arrow), *cycG* ^*HR7*^ mutant larvae float (marked with arrow) in the buoyancy test. In a hs-*CycG* background at ambient temperature, *cycG* ^*HR7*^ mutant larvae subside to the bottom, as also seen for hs-*CycG* larvae. (D') Statistical evaluation of the buoyancy test repeated five times with 10 larvae each. Error bars denote standard deviation. ***p<0.001 according to Student’s T-test. (E-J') Oil Red O staining was performed on larval pelts to visualize lipid droplet accumulation in oenocytes. Larvae were either well-fed with yeast paste (E-J), or starved for amino acids (E'-J'). For a statistical evaluation see S3 Fig. The following genotypes were tested: (E-E') wild type (Wt). (F-F') *cycG* ^*HR7*^ homozygotes. (G) hs-*CycG*; *cycG* ^*HR7*^ (ambient temperature). (H) hs-*CycG* (ambient temperature). (I-J') Overexpression of a control (UAS-*GFP*) or of TSC1/2 specifically in the larval fat body. (I-I') *Adh*-Gal4/+; UAS-*GFP*/+ (Adh::GFP). (J-J') *Adh*-Gal4/+; UAS-*TSC1* UAS-*TSC2* (Adh::TSC1/2). Scale bar: 20 μm.

The distinct weight reduction of *cycG* mutant flies suggested a defect in metabolic homeostasis. Weight is a parameter that is directly correlated with food intake and metabolism. However, the ingestion of *cycG* mutant animals appeared normal as judged by the intake of colored yeast paste (Fig 2A). When exposed to starvation stress, *cycG* ^*HR7*^ mutant flies had a reduced life span compared to the wild type control (Fig 2A' and 2A''). Moreover, *cycG* ^*HR7*^ mutant larval fat bodies displayed an aggregation of lipid droplets similar to starved controls (Fig 2B–2B''). This phenotype has been described before as a result of amino acid withdrawal and likewise loss of TOR and can be taken as an early evidence of fat mobilization for energy consumption [20]. We therefore determined the proportion of lipid and protein in the *cycG* mutants compared to control larvae of the same developmental stage. We noted a significant shift in favor of the triacylglycerol (TAG) level in *cycG* mutant larvae, suggesting an elevated level of stored fat (Fig 2C and 2C' and S2A Fig). Fat accumulation was further confirmed with a buoyancy-based assay [32]. Whereas wild type larvae sink in a 10% sucrose solution, *cycG* mutant larvae float due to their higher fat content (Fig 2D and 2D' and S2B and S2B' Fig). The metabolic defect in the *cycG* ^*HR7*^ mutant larvae was rescued by low level expression of the hs-*CycG* construct, which on its own was indistinguishable from the control, emphasizing the specific requirement of CycG for a normal fat metabolism (Fig 2C and 2D').

During poor nutritional conditions TAG is mobilized from the larval fat body and the free fatty acids are delivered to the larval oenocytes [33]-[35]. Oenocytes are hepatocyte-like cells that are clustered underneath the lateral epidermis of the larva [33]. To test lipid release in *cycG* mutants, we examined lipid accumulation in the oenocytes under conditions of feeding and starvation (i.e. amino acid deprivation) [33]. Well fed control larvae show little lipid accumulation in the oenocytes (Fig 2E and 2H), yet an aggregation of lipid droplets is observed after a 14 hours fasting period (Fig 2E'). Oenocytes of *cycG* mutant larvae accumulated numerous lipid droplets already under normal feeding conditions (Fig 2F and S2C–S2F Fig), which was rescued by the hs-*CycG* background at ambient temperature (Fig 2G), but which was hardly increased under conditions of starvation (Fig 2F'). The observed differences were highly significant (S3 Fig). In summary, *cycG* mutants display a starvation phenotype even under normal feeding conditions suggesting a defect in the regulation of lipid mobilization from the fat body. The influence of TOR activity on lipid metabolism is well established [35]. For example, a downregulation of the TORC1 signal by overexpression of the negative regulator TSC1/2 in the fat body has been shown to provoke a marked lipid droplet accumulation in the oenocytes of well fed larvae [33] (Fig 2I and 2J'). We noted that the loss of CycG had a very similar effect as the overexpression of TSC1/2 (compare Fig 2F and 2J), further supporting a link between CycG and the InR/TOR signaling pathway.

### *cycG* mutants display defects in InR/TOR signaling activity

**Fig 3:**
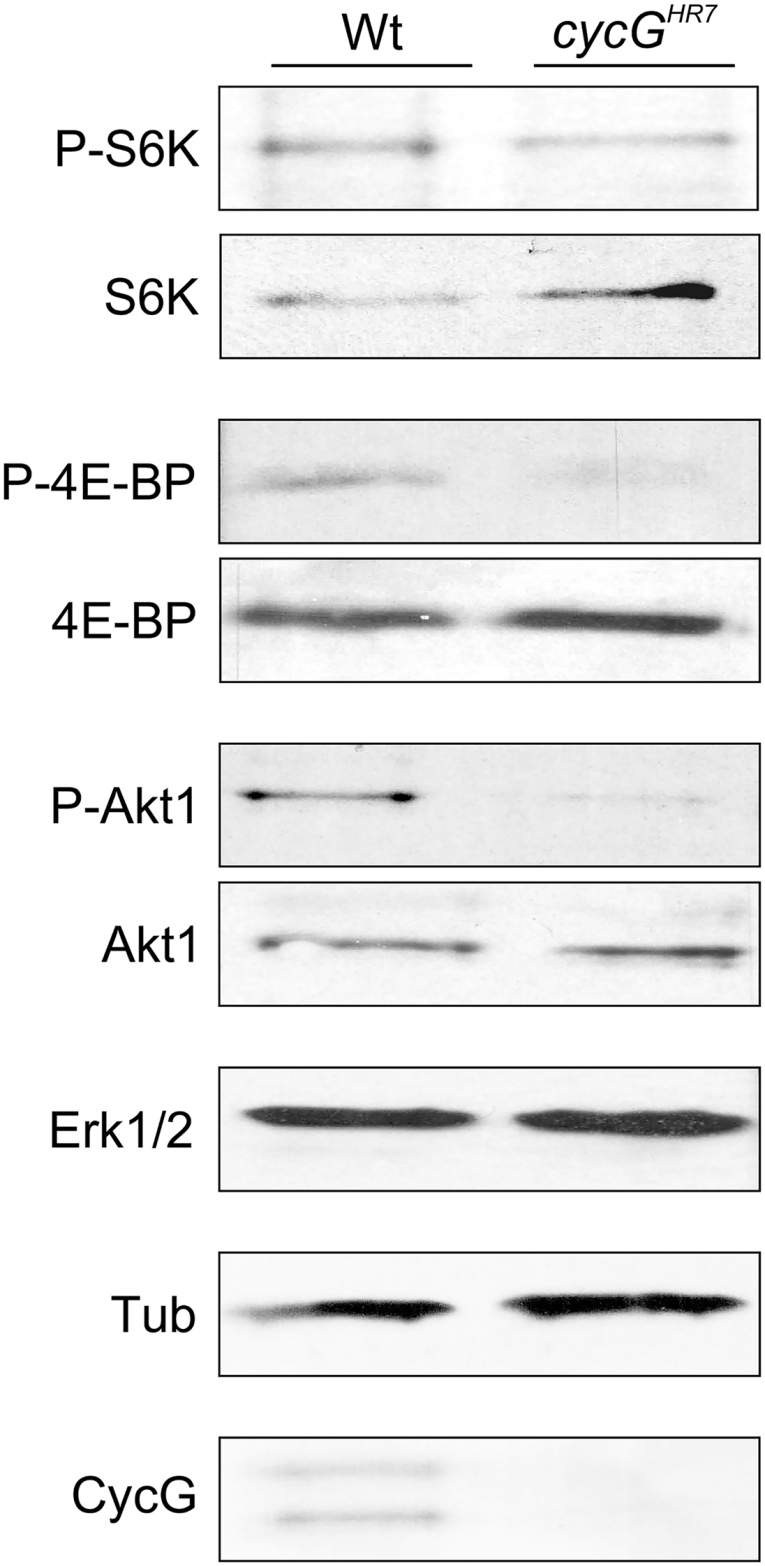
Reduced phosphorylation levels of InR/TOR targets in *cycG* ^*HR7*^ mutants. The TOR targets S6 kinase (S6K) and 4E-BP, as well as Akt1 kinase, were assayed for their phospho-status (P, phosphorylated) by Western blotting using the respective antibodies as indicated. As a control, the blots were probed for the presence and absence of CycG, respectively, and to determine equal loading, for either Erk1/2 or beta-Tubulin, as tubulin levels might change when InR signaling is influenced [15].

The primary downstream targets of TORC1 are S6 kinase (S6K) and elF-4E binding protein (4E-BP) (for review [22], [23]). The growth defects of the *cycG* mutants prompted us to analyze the phosphorylation level of S6K and 4E-BP in protein extracts from *cycG* ^*HR7*^ and wild type control flies (Fig 3): as expected for a positive role of CycG in TORC1 signaling, the level of the phosphorylated isoform was each decreased in the mutant (Fig 3). We further addressed the phosphorylation status of the kinase Akt1, which is at the point of intersection between InR and TOR signaling cascades, activating the latter (for review [2], [8]). Interestingly, phosphorylation levels of Akt1 were also reduced in the *cycG* mutants (Fig 3 and S4A Fig), and rescued to normal in the hs-*CycG* background at ambient temperature, which displayed normal levels on its own (S4A Fig).

Due to its impact on energy homeostasis and cell growth, loss of TOR in *Drosophila* affects multiple tissues and organs. For example, *TOR* mutants display characteristic defects in endoreplication, e.g. of cells in the salivary glands, accompanied by a reduction of Cyclin E levels that regulate G1/S phase entry in mitotic and endoreplicative tissues [20]. In fact, the salivary glands and their polytene nuclei were smaller in *cycG* ^*HR7*^ mutant larvae compared to control, indicative of a reduced ploidy (S4B–S4D Fig). In addition, we also observed a reduced level of Cyclin E protein in the *cycG* ^*HR7*^ mutant compared to wild type larvae of the same developmental stage (S4E Fig). In summary, *cycG* mutants phenocopy a reduced TORC1 activity, which can be explained at the molecular level by a requirement of CycG for full Akt1 activity, and hence, TOR activation.

### CycG influences dILP abundance in the brain

**Fig 4:**
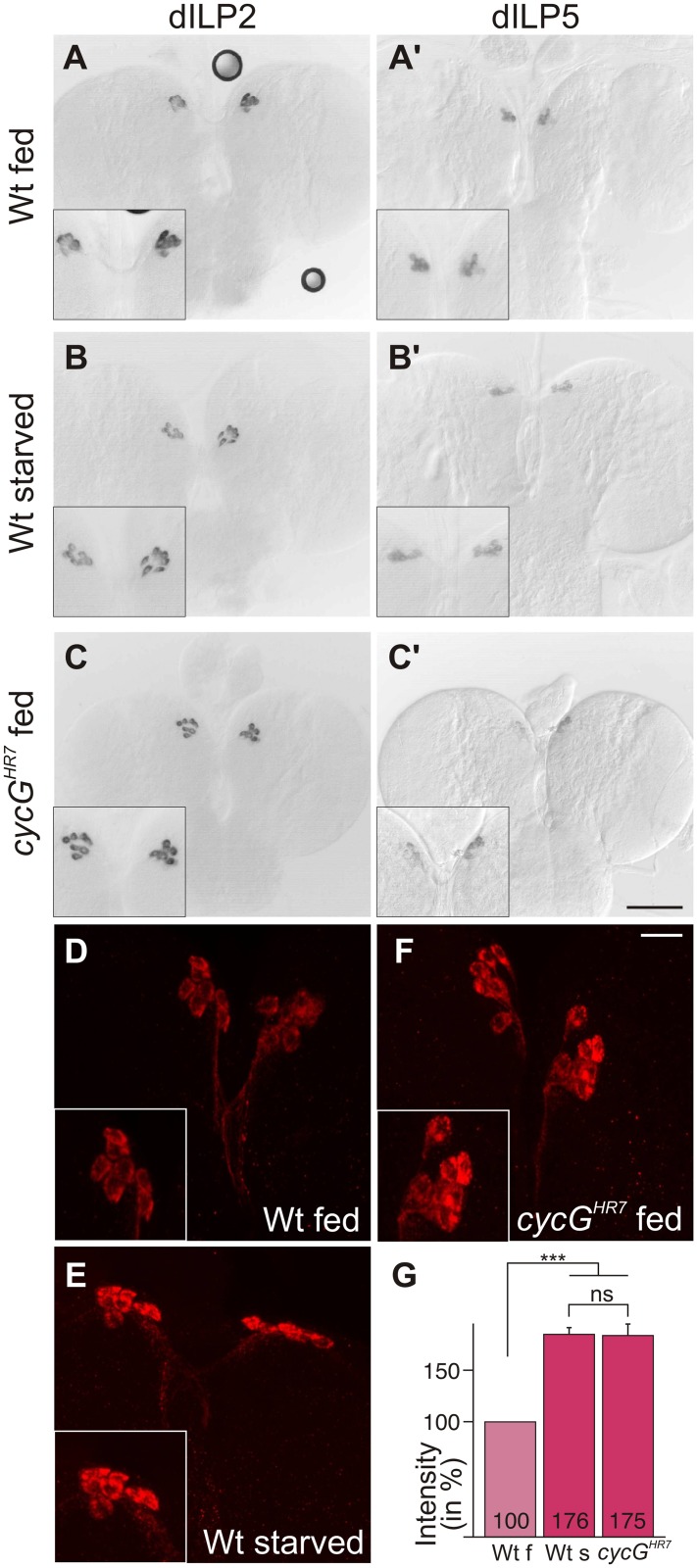
Disturbed dILP accumulation in *cycG* ^*HR7*^ mutant larvae. (A-C) Expression of *dILP2* mRNA in the IPCs of larval brains was examined in fed (A) and starved (B) wild type (Wt) and fed *cycG* ^*HR7*^ mutant larvae (C). There was no apparent difference. (A’-C’) In well-fed wild type larvae (A’), mRNA expression of *dILP5* is notably stronger than in starved larvae (B’) [34]. In the well-fed *cycG* ^*HR7*^ mutant larvae, *dILP5* expression was very weak (C’). (D-F) Under starvation conditions, DILP2 protein accumulates in IPC bodies (compare D, Wt fed with E, WT starved). A similar enrichment was observed in well-fed *cycG* ^*HR7*^ mutant larvae (F). Scale bars: (A-C') 50 μm; (D-F) 10 μm. Insets in A-F show enlargements of IPCs. (G) Staining intensity of IPC groups was measured in fed wild type (Wt f) [n = 10], starved wild type (Wt s) [n = 6] and fed *cycG* ^*HR7*^ homozygotes [n = 18]; fed wild type was taken as 100%. Note similar levels of dILP2 enrichment in starved wild type and fed *cycG* mutant larvae. Error bars denote standard deviation. ***p<0.001; ns: not significant according to Student’s T-test.

TORC1 activity in the larval fat body is required for the release of *Drosophila* Insulin-like peptides (dILPs) from specialized neurosecretory cells in the brain called insulin-producing cells (IPCs) [4]. Four of the eight known *dILP* genes (*dILP1*, *2*, *3* and *5*) are specifically expressed in IPCs [3], [5]. Interestingly, only IPC-derived transcription of *dILP3* and *dILP5* is sensitive to food deprivation, whereas *dILP2* transcription remains unchanged [36]. Expression of *dILP2* and *dILP5* was monitored in the brains of *cycG* ^*HR7*^ mutant third instar larvae under fed conditions. In contrast to *dILP2* (Fig 4A–4C), *dILP5* expression was reduced in the *cycG* ^*HR7*^ mutant comparable to the level observed in starved control animals (Fig 4A'-4C'). Moreover, it was reported that the production and secretion of dILP2 and dILP5 peptides are also controlled post-transcriptionally by nutritional inputs [4]. Under normal fed conditions, both proteins are evenly distributed within the IPC cell body and its axons. Upon starvation, a strong accumulation of dILP protein is observed in the IPC cell body and the axonal termini [4] (see also Fig 4D and 4E for controls). Although this analysis does not allow discriminating between insulin production and secretion, it does reveal changes in insulin dynamics [37]. In accordance with the starvation phenotype of the *cycG* mutants, we detected a strong increase in dILP2 protein labeling similar to that of the IPCs in starved wild type larval brains (compare Fig 4E and 4F). Compared to well-fed larvae, the signal intensity was nearly doubled in starved wild type larvae as well as in well-fed *cycG* ^*HR7*^ mutant larvae (Fig 4G). Ablation of dILP producing cells is correlated with a reduced egg production, linking dILP activity directly to fertility [38]. As expected by the perturbed dILP2 abundance in IPCs, *cycG* ^*HR7*^ mutant females laid significantly less eggs per day, reaching only two thirds of control females (S4F Fig). This phenotype was normalized in the hs-*CycG* background at ambient temperature (S4F Fig). Together, these observations indicate that CycG is required for the endocrine control of dILP production or secretion that is regulated by TOR activity.

### An elevated level of Akt1 ameliorates *cycG* mutant defects

**Fig 5:**
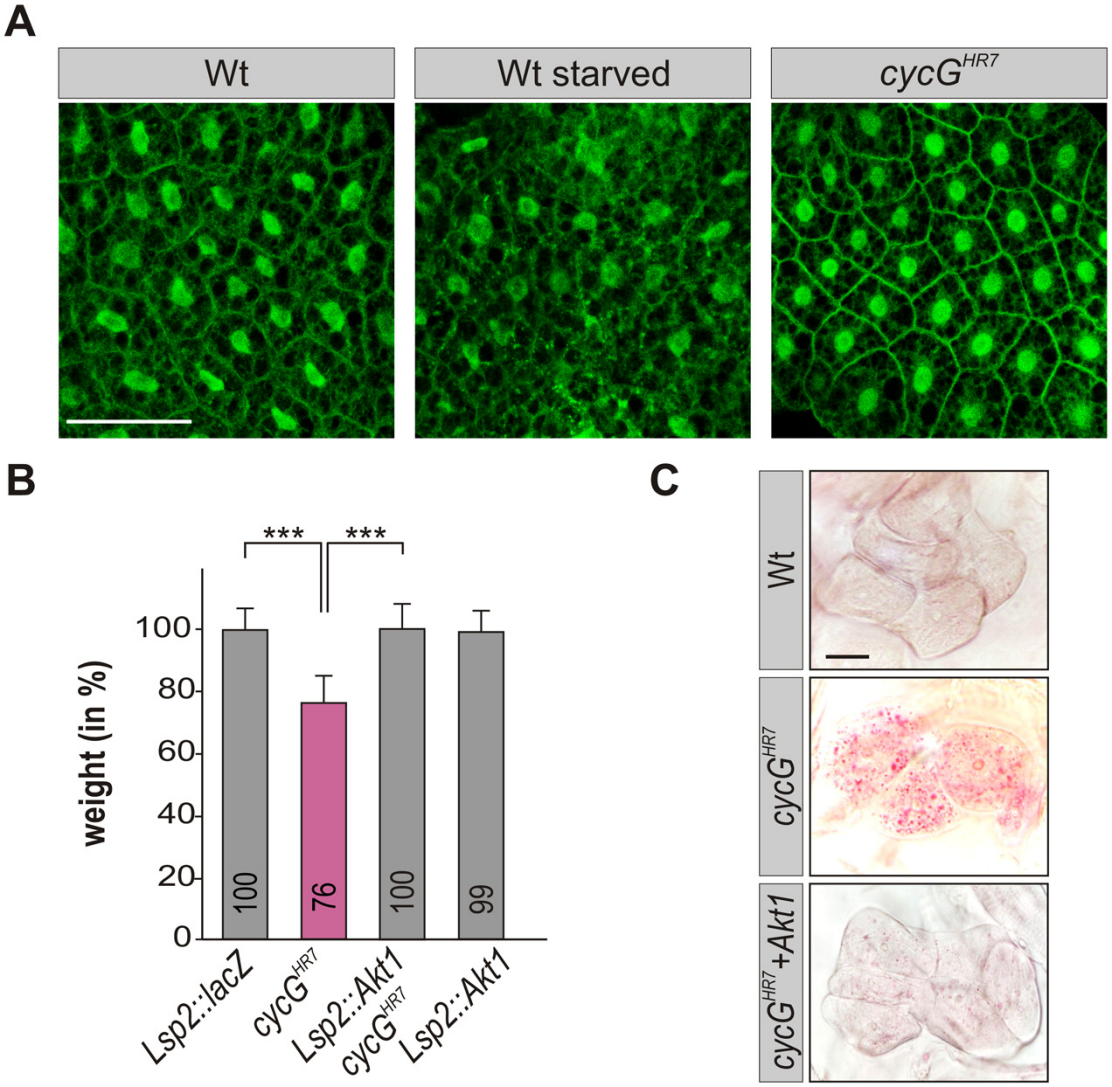
*CycG* acts genetically downstream of PI3K92E. (A) *tGPH* localization in fat body cells of either well-fed or starved wild type (*tGPH/Cy*O; WT, left panels) or of well-fed *cycG* ^*HR7*^mutant larvae (*tGPH/Cy*O; *cycG* ^*HR7*^ */cycG* ^*HR7*^; right panel) is shown. Membrane localization of *tGPH*, an indicator of PI3K92E activity, was not apparently altered in *cycG* ^*HR7*^ mutant larvae compared to well-fed wild type. Scale bar: 100 μm. (B) Overexpression of Akt1 specifically in the larval fat body using the *Lsp2*-Gal4 driver rescues the reduced body weight of homozygous *cycG* ^*HR7*^ mutants. Genotypes are: *Lsp2*-Gal4/UAS-*lacZ*. *cycG* ^*HR7*^ */cycG* ^*HR7*^. UAS-*Akt1*/+, *Lsp2*-Gal4 *cycG* ^*HR7*^ */cycG* ^*HR7*^. UAS-*Akt1*/+, *Lsp2*-Gal4/+. Error bars denote standard deviation [n = 100 per genotype]. ***p<0.001 according to Student’s T-test. (C) Lipid droplet accumulation in the oenocytes of the *cycG* ^*HR7*^ mutant is largely normalized by the overexpression of Akt1 within the larval fat body. Genotypes are: wild type (upper panel); homozygous *cycG* ^*HR7*^ (middle); UAS-*Akt1*/+, *Lsp2*-Gal4 *cycG* ^*HR7*^ */ cycG* ^*HR7*^ (lower panel). Scale bar: 20 μm. For a statistical evaluation see S3 Fig.

As the growth and metabolic deficits in *cycG* mutants reveal an impairment of InR/TORC1 signaling activity, we tested the potential role of CycG in this network. One of the first effectors of the activated InR is PI3K92E whose activity was monitored with the *tGPH* reporter in *cycG* larval tissues [39]. This reporter expresses GFP fused to a pleckstrin-homology domain (GPH) that is recruited to the plasma membrane upon PI3K activation [39]. We compared fat body cells from early third instar wild type larvae under fed and starved conditions with those of fed *cycG* ^*HR7*^ mutant larvae (Fig 5A). In agreement with the published data, *tGPH* accumulated little along the fat body cell membranes in starved larvae (Fig 5A) [39]. In the *cycG* ^*HR7*^ mutants, the *tGPH* reporter highlighted the membranes as strongly as in the well-fed wild type, indicating a normal threshold of PI3K92E activity in the absence of CycG (Fig 5A). The next factor downstream of PI3K, Akt1 shows a reduced phosphorylation level in *cycG* ^*HR7*^ mutants (Fig 3). We hence studied the ability of Akt1 to rescue the growth defects of the *cycG* mutant by genetic epistasis experiments. To this end, Akt1 was specifically induced in the larval fat body of *cycG* ^*HR7*^ mutant larvae, resulting in normal weight animals (Fig 5B). Furthermore, lipid droplet accumulation in oenocytes of *cycG* ^*HR7*^ mutant larvae was considerably improved by the fat body specific expression of Akt1, commuting to a more wild type level (Fig 5C and S3 Fig). These data show that activation of InR/TORC1 signaling at the level of Akt1 is sufficient to counteract the starvation phenotype of *cycG* mutants.

### Mutants in PP2A-B' subunit *widerborst (wdb)* rescue *cycG* mutant phenotypes

**Fig 6:**
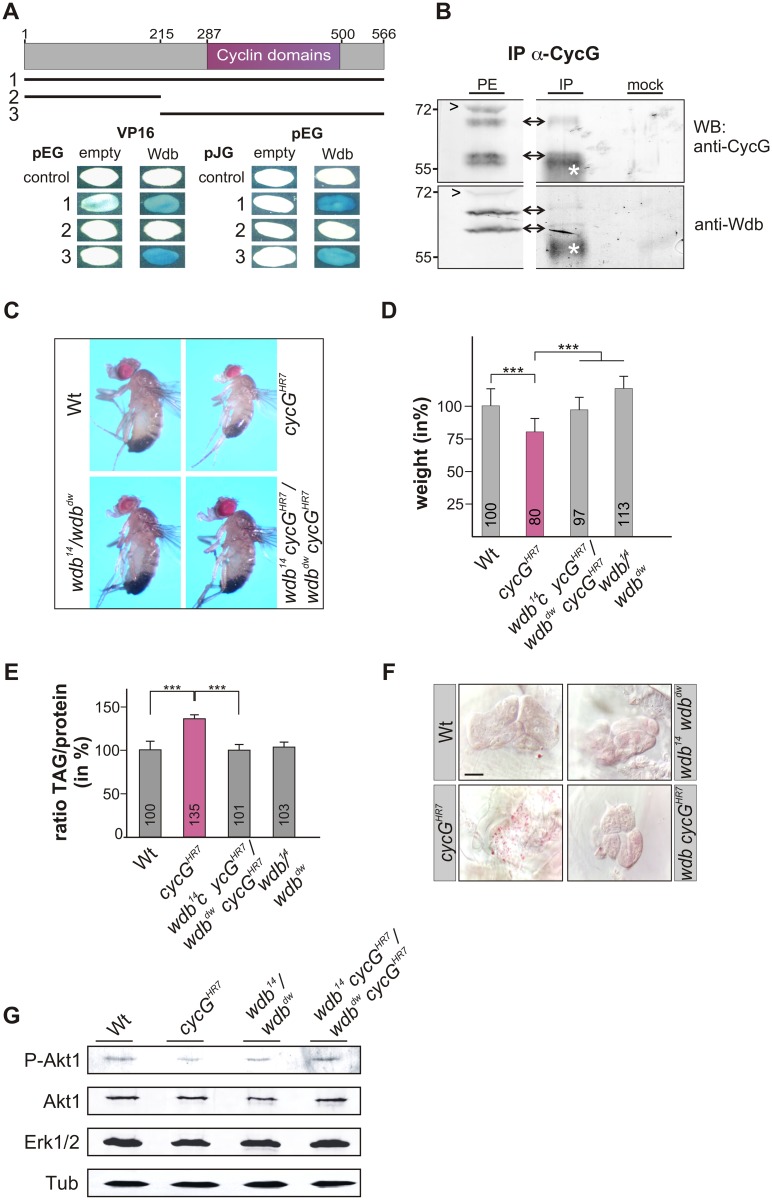
Interaction of CycG and Wdb. (A) Schematic representation of the CycG protein. Three constructs were used in a yeast two-hybrid assay, the full length protein (1, AS 1–566), the N-terminal region (2, AS 1–215) or the C-terminal region including the conserved Cyclin domains shown in magenta (3, AS 215–566). The interaction assay was done in both orientations, with CycG cloned into pEG vector or pJG vector and full length Wdb in pEG or VP16 vector. Empty vectors served as negative controls. Positive interaction is visualized by the blue staining of yeast colonies. (B) CycG and Wdb proteins can be co-precipitated *in vivo*. CycG proteins were immunoprecipitated (IP) from embryonic extracts using guinea pig anti-CycG antibodies and were probed with anti-CycG (upper box, arrows) or anti-Wdb antibodies (lower box, arrows), both from rat. The input lane contained 25% of the protein extract (PE) used for the IP. Arrowheads point to unspecific signals. Guinea-pig preimmune serum was used as mock control. The asterisks label unspecific IgG signals. Blots were cut to allow for exposure adjustment of the input. Size is given in kDa. (C) *wdb cycG* double mutant animals retain normal body size. In contrast to the small homozygous *cycG* ^*HR7*^ males, *wdb cycG* double mutant animals (*wdb* ^*14*^ *cycG* ^*HR7*^/*wdb* ^*dw*^ *cycG* ^*HR7*^) are more similar to wild type (Wt) or trans-heterozygous *wdb* mutants (*wdb* ^*14*^ */ wdb* ^*dw*^ *)* with regard to size. (D) The weight deficit of homozygous *cycG* mutants is significantly rescued in a *wdb* mutant background, relative to wild type (Wt, taken as 100%). Depicted are wild type (Wt), *cycG* ^*HR7*^ homozygous mutant, *wdb* ^*14*^ *cycG* ^*HR7*^/*wdb* ^*dw*^ *cycG* ^*HR7*^ and *wdb* ^*14*^ */ wdb* ^*dw*^ flies. Error bars denote standard deviation [n = 100 per genotype]. ***p<0.001 according to Student’s T-test. (E) Shown are relative larval TAG levels, normalized to total body protein content. Wild type (Wt) levels were taken as 100%. The highly increased TAG levels of the *cycG* ^*HR7*^ mutants are significantly reduced in *wdb cycG* double mutants to nearly wild type levels. Error bars denote standard deviation [n≥3 experiments per genotype]. ***p<0.001 according to Student’s T-test. (F) Lipid droplets are largely absent from oenocytes in well fed wild type control animals (Wt) in contrast to homozygous *cycG* ^*HR7*^. Loss of *wdb* activity largely normalized the lipid accumulation in the *cycG* ^*HR7*^ mutant. Genotypes are: Wild type (Wt), *cycG* ^*HR7*^ homozygous; *wdb* ^*14*^ */ wdb* ^*dw*^ trans-heterozygous; *wdb* ^*14*^ *cycG* ^*HR7*^/*wdb* ^*dw*^ *cycG* ^*HR7*^double mutant (*wdb cycG* ^*HR7*^). Scale bar: 20 μm. (G) The *wdb cycG* double mutants show nearly normal Akt1 phosphorylation levels. Protein extracts from heads of wild type (Wt), *cycG* ^*HR7*^, *wdb* ^*14*^ */ wdb* ^*dw*^ and *wdb* ^*14*^ *cycG* ^*HR7*^/*wdb* ^*dw*^ *cycG* ^*HR7*^ mutants were probed with antibodies detecting either the unphosphorylated (Akt) or phosphorylated form (P-Akt) of Akt1. Erk1/2 and beta-Tubulin (Tub) served as loading controls.

*Drosophila* Widerborst (Wdb) is a B' subunit of the protein phosphatase 2A (PP2A) (for review: [40]). Wdb acts as a negative regulator in the InR/TOR network by targeting PP2A to dephosphorylate Akt1 [18]. Both mammalian CycG homologues, CycG1 and CycG2, interact directly with several B' subunits of PP2A, acting as specificity factors [41], [42]. A likewise direct molecular interaction of CycG and Wdb has been predicted in *Drosophila* [27], [28], which we confirmed in a yeast two-hybrid assay, showing that it involves the conserved cyclin domains (Fig 6A). Moreover, Wdb and CycG were co-precipitated from embryonic extracts, indicating *in vivo* complexes including the two proteins (Fig 6B and S5A Fig).

To determine whether the growth and metabolic defects observed in *cycG* mutants might be due to a deregulated PP2A activity, we first assayed the size and weight of *wdb cycG* double mutant larvae and adults. To this end, the two *wdb* alleles *wdb* ^*14*^ and *wdb* ^*dw*^ were used, which are lethal in homozygosis but viable in the trans combination [43]. Each allele was recombined with the *cycG* ^*HR7*^ allele to generate the double mutant heteroallelic combination. We found that *wdb cycG* double mutant larvae and adults showed a nearly wild type size and weight (Fig 6C and 6D and S5B and S5B' Fig). Accordingly, lipid storage defects of the *wdb cycG* double mutant larvae were likewise normalized, i.e. TAG-levels, specific weight and lipid droplet accumulation in the oenocytes were similar to wild type (Fig 6E and 6F and S5C and S5C' Fig). Finally, the abundance of phosphorylated Akt1 in the *wdb cycG* double mutants was similar to the control and no longer diminished compared to the *cycG* ^*HR7*^ homozygotes (Fig 6G).

### CycG negatively influences the binding of Wdb and Akt1

The remarkably diverse and cell type specific functions of Akt1 in the context of InR/TOR signaling have recently been attributed to the existence of different subcellular pools of activated Akt1 kinase that control different cellular processes [44]. For example, whereas activated Akt1 is predominantly found at the apical membrane of *Drosophila* eye tissue, it is mostly cytoplasmic in the *Drosophila* ovary, where it regulates the lipid metabolism in nurse and follicle cells [18], [44], [45]. This specific ovarian function of Akt1 is under the control of Wdb, selectively modulating the levels of cytoplasmic phosphorylated Akt1 and thereby lipid droplet size in ovarian cells [18]. Accordingly, Wdb and Akt1 physically interact in the ovary, whereas no interaction was observed in larval tissue [18].

**Fig 7:**
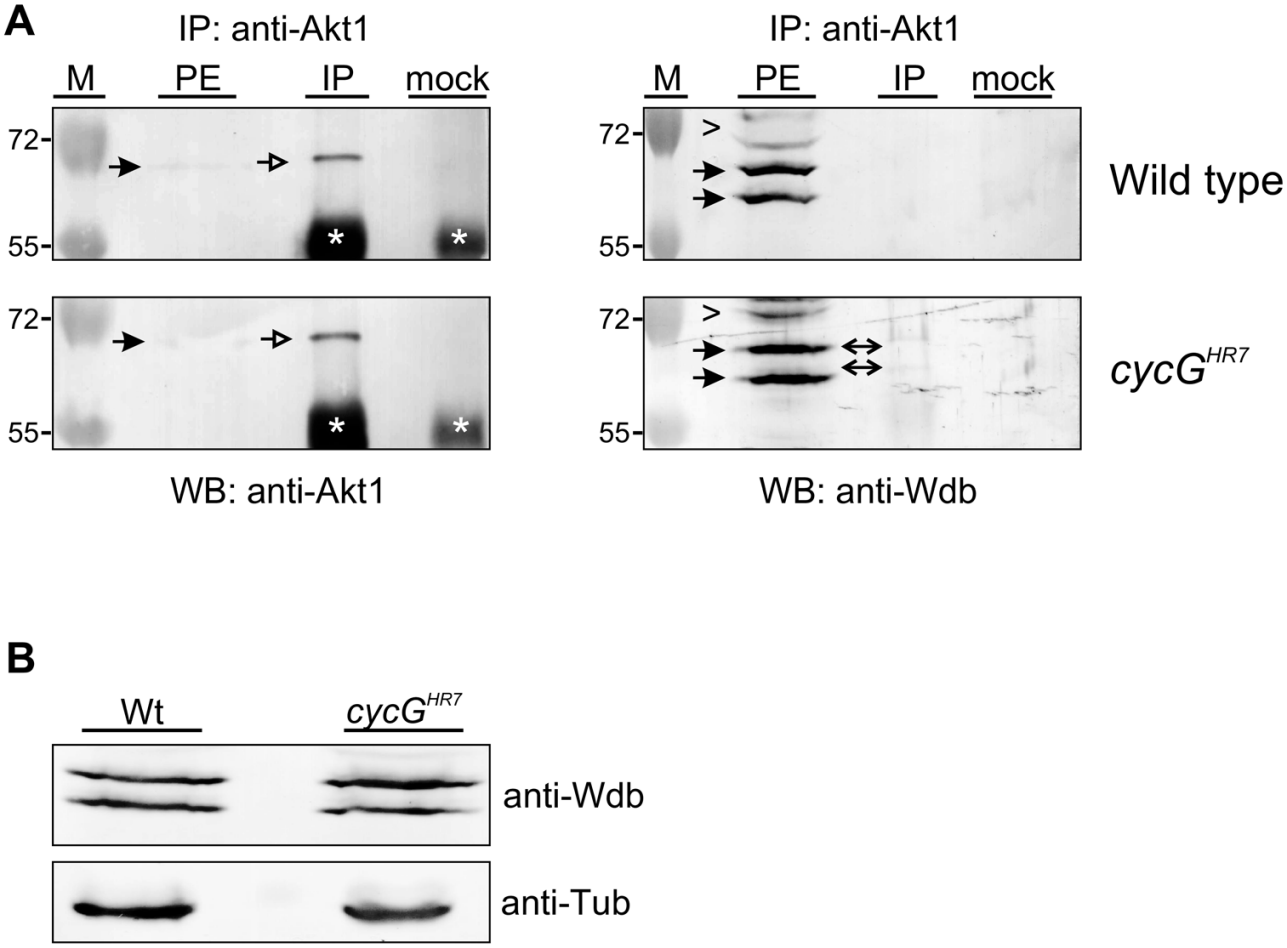
Influence of CycG on Akt1-Wdb binding. (A) Akt1 and Wdb proteins interact *in vivo* in *cycG* ^*HR7*^ mutants but not in the wild type. Akt1 proteins were immunoprecipitated (IP) from head extracts of either wild type or *cycG* ^*HR7*^ mutant animals and probed with anti-Akt1 (left panels, arrows) or anti-Wdb antibodies (right panels, arrows) as indicated. The input lane contained 25% of the protein extract (PE) used for the IP. Despite low Akt1 abundance in head extracts, Akt1 was robustly immunoprecipitated in wild type or *cycG* ^*HR7*^ mutants (left panels, open arrows), whereas Wdb protein was only co-precipitated in the mutant (right panels, double headed arrows). Arrowheads point to unspecific signals. Unrelated serum was used as mock control. The asterisks label unspecific IgG signals. M, size standard in kDa. (B) Wdb proteins were detected by Western blotting in head extracts of either *cycG* ^*HR7*^mutants or wild type (Wt) control as indicated. Beta-Tubulin (Tub) served as loading control.

**Fig 8:**
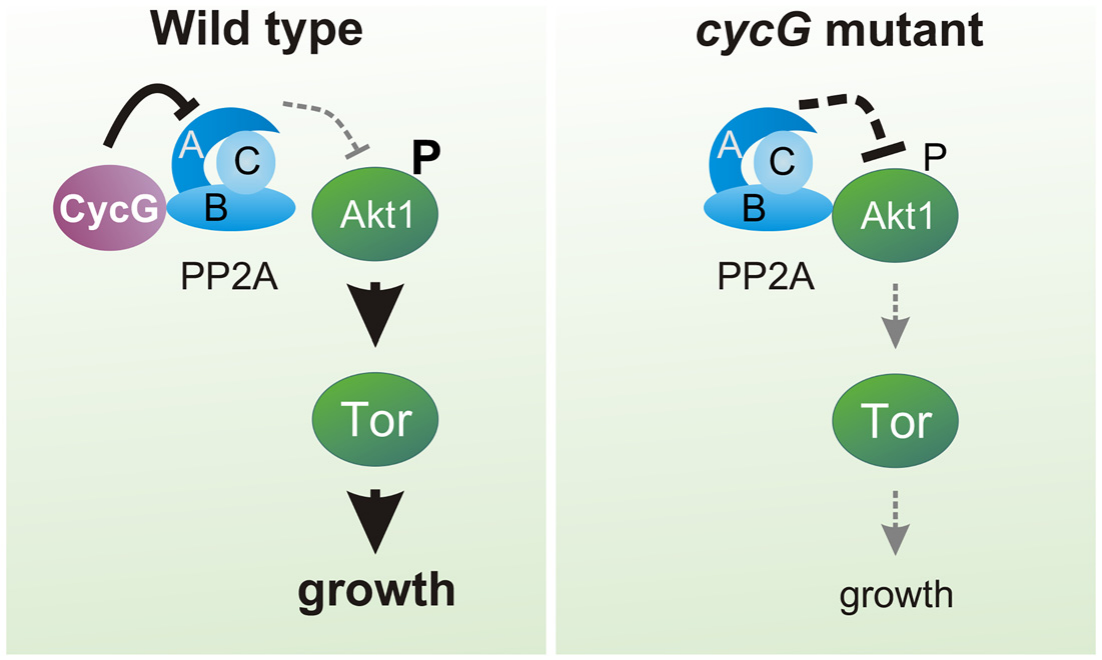
Model of CycG function. In a wild type fly, CycG interacts with a B' subunit of PP2A affecting its binding of Akt1. This leads to a balanced level of InR/TOR signaling mediated growth (left). In *cycG* deficient flies, Wdb-Akt1 binding is favored and phosphorylation of the central kinase Akt1 is decreased. As a consequence, TOR mediated growth is impaired (right).

Because both larval and adult phenotypes of *cycG* mutants depend on Wdb activity (Fig 6 and S5 Fig), we asked whether CycG may influence the physical interaction of Wdb and Akt1. Indeed we observed a robust interaction of Akt1 and Wdb in head extracts from *cycG* ^*HR7*^ mutant animals in contrast to control animals (Fig 7A), implying an involvement of CycG in the regulation of Akt1/Wdb binding. Both protein species of Wdb co-precipitated with Akt1 (Fig 7A), indicating that the binding of Akt1 is not restricted to the higher molecular weight species of Wdb as previously reported [18]. Accordingly, no influence of CycG on the relative abundance of the two protein species of Wdb was detected comparing wild type and *cycG* ^*HR7*^ protein extracts (Fig 7B). Overall our data point to a causal link between CycG and PP2A activity in the regulation of growth and metabolism in *Drosophila* at the level of Akt1 (Fig 8): the presence of CycG may disfavor binding of PP2A-B’ to Akt1, which is facilitated in its absence, resulting in a decrease of phosphorylated, i.e. activated Akt1.

## Discussion

In this study, we have analyzed the role of Cyclin G in growth regulation and metabolism of *Drosophila*. We made use of two different *cycG* null mutant alleles, thereby allowing us to follow the developmental consequences resulting from the absence of *cycG* gene activity instead of drawing conclusions from overexpression or RNAi experiments. Misexpression studies initially raised the assumption that CycG negatively regulated cell growth and cell proliferation in *Drosophila* [26]. Our results now indicate that CycG is required for normal growth, affecting both cell size and cell number. In fact, clonal analysis revealed a cell autonomous requirement of CycG not only in the wing but also the eye anlagen (Fig 1F and 1F’). In addition, the *cycG* null mutants show signs of metabolic disorder. We provide evidence that CycG facilitates InR/TORC1 mediated growth regulation via PP2A, thereby helping to sustain nutrient dependent growth in *Drosophila* (Fig 8).

### The multiple roles of CycG in *Drosophila*

*Drosophila* CycG appears to have extraordinarily diverse roles. It has been involved in epigenetic regulation of homeotic gene activity, in cell cycle regulation, developmental stability and in DNA repair [24], [26], [46], [47], and now also in metabolic homeostasis. Our current work confirmed molecular interactions between CycG and Wdb proteins *in vivo* that had been predicted from genome-wide proteome analyses *in vitro* [27], [28]. Interestingly, similar molecular interactions have been described before for mammalian CycG1 and CycG2: both proteins interact with several B' subunits, thereby mediating the recruitment of PP2A to its different substrates [41], [42]. In contrast to mammals, the genetic relationship between CycG and PP2A is antagonistic in *Drosophila* as a reduction of PP2A activity ameliorates the consequences of CycG loss. The *cycG* mutation could be formally explained by a gain of PP2A activity. It is tempting to speculate that the diversity of CycG functions results from a regulation of PP2A by CycG. PP2A affects a plethora of developmental and cellular processes, hence, pleiotropy is expected in case of its misregulation (for review [40], [48]). Most likely, this hypothesis is too simplified. For example, loss of *cycG* in the female germ line results in an increase of phosphorylated H2Av (gamma-H2Av) [24], a known target of PP2A activity [49]. One might have expected a reduced amount of gamma-H2Av if loss of CycG equated with a gain in PP2A activity. Instead, we have shown that CycG is found in a protein complex together with Rad9 and BRCA2 that primarily acts in the sensing of DNA double strand breaks [24]. The importance of *Drosophila* CycG in DNA double strand break repair is reminiscent of functions described for mammalian CycG proteins: albeit CycG1 and CycG2 mutant mice are viable and healthy, they are both sensitive to DNA damaging reagents [50], [51]. Moreover, upregulation of CycG2 was involved in the activation of Chk2 and in damage induced G_2_/M cell cycle arrest, i.e. in DNA damage response in mammals as well [51]. Whether the other phenotypes and interactions reported for *Drosophila* CycG are linked to the regulation of PP2A remains to be addressed in more detail.

### *Drosophila* CycG is required for InR/TOR-mediated growth and metabolism

The *cycG* mutants display several phenotypic characteristics of a diminished TORC1 signaling activity [20], [33], including weight reduction, a reduced egg laying rate, impaired endoreplication and a general increase in lipid mobilization.

Moreover, CycG activity promotes phosphorylation of the primary TORC1 targets, i.e. S6K and 4E-BP. In contrast to *TOR* mutants, however, *cycG* mutants are viable, implying that CycG facilitates InR/TOR signaling rather than being an essential factor. Overall, *cycG* mutant flies show typical signs of nutritional starvation distress even under normal food conditions, suggesting a problem in their capacity to take up food and/or to sense and utilize the food. This defect is not due to a general inability of the animal to grasp the feed, but instead reflects a defect in coordinating the energy status with the regulation of systemic growth. As dILP accumulation in the brain is altered in *cycG* mutants, we know that the signals transmitted from the nutritional sensor fat body must be disturbed. The fact, that we can strongly ameliorate the growth defects of *cycG* mutants by an induction of Akt1 specifically in the fat body rules out a function of CycG in the endocrine signal emanating from the fat body. Instead, all of our data indicate that CycG acts genetically at the level of Akt1, thereby controlling TOR signaling activity (Fig 8).

Akt1 is negatively regulated by PP2A (for review [48]), supporting a model whereby CycG exerts its positive input on Akt1 via an inhibition of PP2A. In accordance, mutations in *wdb* efficiently rescue the growth and metabolic defects observed in *cycG* mutants. Likewise a downregulation of Wdb ameliorates the weight deficits resulting from a loss of Akt1 activity [18]. In *Drosophila*, Wdb acts as a tissue-specific negative regulator of Akt1: it modulates lipid metabolism in the ovary as a result of a direct interaction with Akt1, whereas no such influence was seen in eye tissue [18]. We have shown that Wdb-Akt1 binding in the adult head is favored in the absence of CycG, i.e. CycG is able to influence the interaction between Wdb and Akt1 presumably by its direct binding to Wdb. A consequence of CycG loss may be the enhanced binding of PP2A to Akt1 and an enforced dephosphorylation of Akt1, resulting in the inhibition of downstream TOR signaling activity and affecting lipid metabolism and growth (Fig 8). Moreover, the second B'-subunit of *Drosophila* PP2A (also called Well rounded, Wrd) is involved in the negative regulation of the S6K [15]. Assuming a molecular interaction of Wrd and CycG, a likewise regulatory input of CycG on PP2A containing the Wrd B'-subunit is conceivable. In this case, CycG might influence S6K activity as well, having a regulatory input on InR/TOR signaling also downstream of TORC1. This scenario is complicated by the negative feed back regulation of InR signaling by S6K and of Akt1 by TORC1 [52]. Circular regulation of InR/TOR signaling has been described at several levels, implementing a tight control of dietary signals and growth but complicating genetic analyses [52] (for review [53]).

In conclusion, the identification of CycG as a novel regulator of InR/TOR signaling in *Drosophila* highlights the importance of studying the regulatory network at the Akt1—PP2A nexus. Based on the high conservation of the InR/TOR signaling pathway and its regulation by PP2A, mammalian fat homeostasis is likely to involve similar regulatory control mechanisms to those we have uncovered in *Drosophila*. Our work raises the possibility of an involvement of CycG in InR/TOR-associated diseases that might be modulated by PP2A. A better understanding of the underlying mechanisms could therefore open up avenues for new strategies to fight InR/TOR-associated disorders in the future.

## Materials and Methods

### Fly strains, genetics and mitotic cell clone induction

The *cycG* ^*HR7*^ null allele and the pUASp-*cycG* transgene have been previously described [24]. The generation and verification of the *cycG* ^*eoC*^ allele is described in the supplementary experimental procedures. To generate the hs-*CycG* construct, *cycG* cDNA was cloned as 2.4 kb *EcoR*I/*Kpn*I fragment into the pCasper-hsRX vector [54] and several independent lines were established by P-element mediated germline transformation [55]. For rescue assays an insertion on the second chromosome was used and combined with the *cycG* ^*HR7*^ mutant.

Fat metabolism and growth defects were analyzed with the following fly strains (BL strains were from Bloomington stock center): *Adh*-Gal4 (gift from R. Kühnlein) [39]), *da*-Gal4 (BL8641), *Lsp2*-Gal4 (BL6357); UAS-*Akt1* (BL8191), UAS-*GFP* (BL4776), UAS-*lacZ* (BL8529), UAS-*TSC1* UAS-*TSC2* [56], the *tGPH*-reporter (gift from B. Edgar) [39] and the *wdb* mutant alleles *wdb* ^*14*^/TM6B and *wdb* ^*dw*^/TM6B (both obtained from C. Wilson) [18], [43]. Oregon-R was used as wild type control. Flies were raised at 25°C under non-crowded conditions on standard cornmeal/molasses/agar medium. For amino acid deprivation, third instar larvae were kept on a sugar only diet [20% sucrose, 1% agar in PBS] for 14 hours.

The Flp/FRT system was used to generate *cycG* ^*HR7*^ mutant clones by mitotic recombination [30]. To this end *cycG* ^*HR7*^ was recombined with the FRT82B bearing chromosome (BL2050). Females of the genotype *yw* hsFlp; FRT82B *cycG*/TM6B were mated with FRT82B arm-lacZ/TM6C (BL7369) males. 24–36 hours after egg laying, the animals were subjected to a 30 minute heat shock at 37°C to induce recombination with low frequency. Control clones were generated in parallel with only the FRT82B bearing chromosome. Mutant cell clones are characterized by the loss of lacZ and were analyzed in imaginal discs of third instar larvae. A total number of 30 discs was assayed and compared with control clones.

### Documentation of adults, determination of cell size and number

Adult males, three to five days old, were used for analysis. Dehydrated wings were mounted in Euparal (Roth; Karlsruhe, Germany). Wing area was measured using Image J software (oval selection for total wing size in two measurements that were sampled). Cell number was determined by counting the individual trichoma on the wing blade in three defined 10.000 μm^2^ squares localized in the L4/L5 field next to the posterior cross vein. Subsequently, the total cell number for the determined wing area was calculated, as was cell size. Pictures were assembled using Corel Draw and Corel Photo Paint. Flies and wings were photographed with an ES120 camera (Optronics; Goleta CA, USA) using Pixera Viewfinder software, version 2.0.

To investigate developmental timing, offspring of six parallel *inter se* crosses with the genotype *w* ^*1118*^; *cycG* ^*HR7*^/+ was counted at days 9 to 20. Since the *cycG* ^*HR7*^ mutant carries a mini-white^+^ gene [24], the homozygous *cycG* ^*HR7*^ and the heterozygous *cycG* ^*HR7*^/+ flies could be distinguished by red and orange eye color, respectively, from the white-eyed control siblings.

### Ingestion, starvation assay and amino acid deprivation

As an assay for ingestion, blue-colored yeast paste was offered to larvae as a food source. Food uptake in the gut was visualized by illuminating larvae from the side and taking pictures with a Pixera camera coupled to a Leica stereo-microscope. For the starvation assay, triplicate batches of 15 three days old males of each genotype were transferred to vials containing 1% agarose in PBS only (wet starvation). Mortality rate was determined by counting the number of dead flies every two hours. Third instar larvae were likewise amino acid deprived for two days before dissection.

### Weight measurement, TAG and protein assay, buoyancy test

Newly hatched male flies were transferred to fresh food vials and maintained at 25°C for three days before measurement. Body weight of 100 flies of each genotype was measured individually with a precision scale. Organismal triacylglycerol (TAG) and protein content was quantified using the Pierce BCA Protein determination Kit (Thermo Fisher Scientific; Rockford IL, USA) and the protocol procedure B of the Triglycerid-Assay Kit (Sigma-Aldrich; St. Louis MO, USA). For each genotype, batches of five third instar larvae were homogenized in 150 μl 0.01% Tween in PBS. After incubation at 70°C for 5 minutes the samples were centrifuged for 1 minute at 5000 rpm. The supernatant was transferred to a fresh microtube and after additional centrifugation for 3 minutes at 14000 rpm the cleared lysate was applied in the appropriate assay. A minimum of three independent experiments was performed for each genotype and the results were sampled. A simplified version of the buoyancy-based screen protocol was used [32]: 10 larvae of each genotype were placed in 3 ml of 10% sucrose solution. After gentle mixing and five minutes without agitation, the number of larvae floating at the surface was counted and documented.

### Statistical evaluation

Statistical significance of probes was determined according to Student's T-test (http://www.physics.csbsju.edu/stats/t-test.html) and p-value was scaled accordingly: p>0.05 (not significant, n.s.); p<0.05 (weakly significant; *); p<0.01 (significant; **); p<0.001 (highly significant; ***).

### Tissue staining protocols

Larval brains were dissected in PBS and fixed for 20 minutes in 4% paraformaldehyde. After several washes with PBS plus 0.3% Triton X100 followed by a preincubation step with 4% normal goat serum, rabbit anti-dILP2 antibody (1:800; gift from P. Léopold) [4] was added and incubated over night at 4°C. Imaginal discs were likewise treated, and stained with anti beta-Galactosidase antibodies (1:50; DSHB; Iowa, USA). Secondary antibodies coupled to DTAF or Cy3 were purchased from Jackson ImmunoResearch (Dianova; Hamburg, Germany). The Oil Red O staining of oenocytes was performed exactly as described previously [33]. Larval fat body was dissected from well fed or amino acid deprived third instar larvae in PBS, fixed in 4% paraformaldehyde for 15 min and stained with Nile Red (Sigma-Aldrich; St. Louis MO, USA) at a concentration of 10 μg/ml. As a measurement of PI3K92E activity *in vivo*, the *tGPH* reporter [39] was used and the degree of membrane *tGPH* localization analyzed in early third larval tissues by confocal microscopy. Fluorescently labeled tissues were mounted in Vectashield. Larval brains were analyzed with a Zeiss-ApoTome Axio Imager using AxioVision Software. Imaginal discs and fat bodies were documented with a Bio-Rad MRC1024 confocal microscope coupled to a Zeiss Axiophot using Laser Sharp 2000 software (Carl Zeiss AG; Oberkochen, Germany). Pictures of fat bodies were inversed for better visibility.

### In situ hybridization

In situ hybridization on larval brains was performed with digoxygenin labeled DNA probes of *dILP2* and *dILP5* according to standard protocols [57]. The templates for the probes were generated by PCR using the following primer sets: UP dILP2: GAT CGT AAA GCA ACC TAA GCA GTA A; LP dILP2: ATT CGT AAA GAG TAA CAT GCA ACA A; UP dILP5: GAT CCC AGT TCT CCT GTT CCT GAT C; LP dILP5: TTT CAA GTT TCA AAG CCG TGC ATA T.

### Immunoblots

For each genotype 100 adult heads were homogenized in RIPA I buffer [50 mM Tris-HCl pH 7.5, 150 mM NaCl, 1% Triton X-100, 0.1% SDS, protease inhibitor cocktail (Roche Diagnostic; Basel, Switzerland)] on ice. After centrifugation, loading buffer was added to the supernatant and the probes were loaded on a SDS-PAGE followed by Western blotting. The extracts were probed with guinea pig anti-CycG (1:400) [46]. As loading controls we used anti-beta-Tubulin (1:50) (E7 DSHB; developed by M. Klymkowsky) and anti-Erk1/2 antibodies (1:1000) (Cell Signaling Technology; Danvers MA, USA), as tubulin levels might change when InR signaling is influenced [15]. The amount of total and phosphorylated protein was determined with rabbit anti-4E-BP (1:100, gift from G. Tettweiler) [58], rabbit anti-Akt1 (1:250), rabbit anti-p70 S6 kinase (1:100), rabbit anti-Phospho-Akt (1:250), rabbit anti-Phospho-p70 S6 kinase (1:100) and rabbit anti-Phospho-4E-BP (1:100) (all from Cell Signaling Technology; Danvers MA, USA).

### Yeast two-hybrid studies and co-immunoprecipitations

Full length *wdb* was PCR amplified from cDNA (*LD34343*, obtained from DGRC, Bloomington IN, USA) and cloned as *Bgl*II/*Not*I fragment into pEG and VP16 (*Bam*HI/*Not*I) vectors. pJG-CycG (1–566), GST-CycG (1–215) and GST-CycG (215–566) DNA was a gift from F. Peronnet, France [46]. The CycG subdivision constructs were PCR-amplified using the pJG/GST-CycG constructs as template and further subcloned as *Eco*RI/*Xho*I fragments in either pEG, pJG or VP16 vectors [59], [60]. Protein-protein interaction assays were done according to standard protocols using the Brent two-hybrid system [59]. Protein expression in yeast cells (EGY40: *Mata*, *ura3*, *his3*, *trp1*, *leu2*, GAL) was verified either with mouse anti-HA (1:1000; St. Louis MO, USA), mouse anti-VP16 (1:100; Santa Cruz Biotechnology, Dallas, USA) or rabbit anti-LexA antibodies (1:1000; Bio Acadamia, Osaka, Japan).

CycG or Wdb protein was immuno-precipitated from about 500 embryos (0–24h) using either anti-CycG antibodies or anti-Wdb antibodies (see supporting materials and methods) as described before [46]. Akt1 and Wdb complexes were co-immunoprecipitated from 150 heads each of either wild type or *cycG* ^*HR7*^ homozygous mutant animals using rabbit anti-Akt1 (1:50; Cell Signaling Technology; Danvers MA, USA) and detected with rabbit anti-Akt1 or rat anti-Wdb (see supporting materials and methods).

## Supporting Information

S1 Fig Generation and analysis of the *cycG* ^*eoC*^ mutant. (A) Genomic structure of the *cycG* locus and the neighboring *medea* and *RpL6* genes according to FlyBase (R6.03). Exons are boxed, lighter shades indicate untranslated regions. Only strongly supported transcripts are depicted. The *cycG* ^*eoC*^ allele was generated by 'ends-out' recombination using the indicated fragments Eo1a and Eo1b (see S1 Text), resulting in the deletion of most of the coding region of *cycG*. (B) *cycG* ^*HR7*^ and *cycG* ^*eoC*^ mutants behave as protein null by western blot analyses. No CycG protein can be detected in adult heads of neither *cycG* ^*HR7*^ nor *cycG* ^*eoC*^ homozygotes nor *cycG* ^*HR7*^ */cycG* ^*eoC*^ trans-heterozygotes. In contrast, CycG protein is detected in the heads of wild type (Wt), hs-*CycG*; *cycG* ^*HR7*^ and hs-*CycG* flies at ambient temperature. Beta-Tubulin served as loading control. (C-C') Size and weight comparison of wild type (Wt) and *cycG* homozygous (*cycG* ^*HR7*^ or *cycG* ^*eoC*^) and trans-heterozygous (*cycG* ^*HR7*^/*cycG* ^*eoC*^) mutant larvae at 126 h of development. Note the smaller size (C) and reduced weight (C') of *cycG* mutants in comparison to the wild type, whereas no significant differences are seen amongst the *cycG* mutants. (D-D') Size and weight comparison of wild type (Wt) and *cycG* homozygous (*cycG* ^*HR7*^ or *cycG* ^*eoC*^) and trans-heterozygous (*cycG* ^*HR7*^/*cycG* ^*eoC*^) mutant adult males. Note the smaller size (D) and reduced weight (D') of the *cycG* mutants in comparison to the wild type. In panels C' and D' error bars denote standard deviation [n = 100 each]. *** p<0.001; ns: not significant according to Student’s T-test.
(PDF)

S2 Fig Disturbed fat metabolism in *cycG* mutant animals. (A) All *cycG* mutant combinations show an increase of larval TAG content. Histogram depicting TAG content normalized to protein content; wild type (Wt) levels were taken as 100%. In all panels error bars denote standard deviation [n≥3 experiments]. ***p<0.001; ns: not significant according to Student’s T-test. (B-B') Buoyancy test using *cycG* mutant larvae as indicated. Compared to wild type (Wt), the mutants mostly float (B). Statistical evaluation of the assay repeated five times with ten larvae each (B'). No significant differences were detected between the different mutant combinations according to Student’s T-test; error bars denote standard deviation. (C-F) Comparable lipid droplet accumulation was observed in *cycG* ^*HR7*^, *cycG* ^*eoC*^ and *cycG* ^*HR7*^/ *cycG* ^*eoC*^ mutant larval oenocytes, in contrast to wild type (Wt) control. Scale bar: 20 μm. For a statistical evaluation see S3 Fig.
(PDF)

S3 Fig Quantification of oenocyte staining. Oenocytes of the given genotypes were analysed (s, starved; f, fed). Staining of oenocytes was quantified as percentage of the stained versus the total area using Image J. Error bars denote standard deviation, the number of cells recorded is indicated. ***p<0.001 and n.s., not significant, according to Student’s T-test. In each instance, there is a highly significant difference between oenocytes that are stained by oil-red-O and those that are not.
(PDF)

S4 Fig *cycG* mutants show further signs of reduced InR/TOR activity. (A) Levels of phosphorylated (P-Akt1) vs. unphosphorylated Akt1 protein were detected in western blots of fly head protein extracts. Compared to the wild type (Wt), *cycG* mutant animals (*cycG* ^*HR7*^; *cycG* ^*eoC*^; *cycG* ^*HR7*^/*cycG* ^*eoC*^) exhibit reduced levels of phosphorylated Akt1 protein. The combination hs-*CycG*; *cycG* ^*HR7*^ shows wild type levels of phosphorylated Akt1 protein at ambient temperature, as does hs-*CycG* on its own. Erk1/2 and beta-Tubulin (Tub) were used as loading control. (B-C) Salivary gland nuclei were stained with propidium iodide (red); phalloidin staining (green) outlines the cells. Note large polyploid nuclei in the wild type (B). Salivary gland cells of *cycG* ^*HR7*^ mutants are smaller, as are the nuclei (C). Scale bar: 20 μm. (D) The nuclear diameter was measured in the central focal section of each nucleus [n = 90]. Wild type control was taken as 100%. Error bars denote standard deviation; ***p<0.001 according to Student’s T-test. (E) Western blot using larval protein extracts of wild type (Wt) and *cycG* ^*HR7*^ mutants were probed for CycE protein (*). Erk1/2 and beta-Tubulin (Tub) were used for a loading control. (F) Eggs laid per day in the presence of yeast were counted for wild type (Wt), *cycG* ^*HR7*^ or *cycG* ^*eoC*^ homozygotes, *cycG* ^*HR7*^/*cycG* ^*eoC*^ trans-heterozygotes as well as hs-*CycG*; *cycG* ^*HR7*^ and hs-*CycG* flies at ambient temperature. 10 females each were aged for two days and mated with 5 wild type males. Eggs were counted every 24 hrs for five consecutive days. The experiment was done in triplicate. *cycG* mutant females show a significantly reduced egg laying rate compared to wild type, whereas the *cycG* mutants do not differ among each other. The egg laying defect was rescued in the hs-*CycG* background at ambient temperature. Error bars denote standard deviation. ***p<0.001, ns: not significant according to Student’s T-test.
(PDF)

S5 Fig CycG and Wdb interactions. (A) Proteins immunoprecipitated (IP) from embryonic extracts using guinea pig anti-Wdb antibodies were probed for Wdb (anti-Wdb; upper row, arrows) or CycG (anti-CycG; lower row, arrows) using respective rat antisera. The input lane contained 25% of the protein extract (PE) used for the IP. Guinea-pig preimmune serum was used as mock control. The asterisks label unspecific IgG signals. Blots were cut to allow for exposure adjustment of the input. M, size standard in kDa. (B-B') The size and weight deficit of the homozygous *cycG* ^*HR7*^mutant is significantly rescued in a *wdb* mutant background. B) Size comparison of late third instar larvae (126 h after egg deposition). B') The weight of 100 larvae each was measured and is shown relative to the wild type control, which was taken as 100%. Depicted are wild type (Wt), *cycG* ^*HR7*^ homozygous mutant, *wdb* ^*14*^ *cycG* ^*HR7*^/*wdb* ^*dw*^ *cycG* ^*HR7*^ and *wdb* ^*14*^ */ wdb* ^*dw*^ animals. Error bars denote standard deviation. ***p<0.001 according to Student’s T-test. (C-C') The number of floating *cycG* ^*HR7*^ mutant larvae is strongly reduced in a *wdb* mutant background (C). (C') Statistical evaluation of the assay repeated five times with 10 larvae each. Error bars denote standard deviation. ***p< 0.001 according to Student’s T-test.
(PDF)

S1 Text Supporting Materials and Methods. (DOC)

## References

[1] Martin DE, Hall MN. The expanding TOR signaling network. Curr Opin Cell Biol. 2005; 17: 156–166. 10.1016/j.ceb.2005.02.008 15780592

[2] Teleman AA. Molecular mechanisms of metabolic regulation by insulin in *Drosophila* . Biochem J. 2010; 425: 13–26. 10.1042/BJ20091181 20001959

[3] Brogiolo W, Stocker H, Ikeya T, Rintelen F, Fernandez R, Hafen E. An evolutionarily conserved function of the *Drosophila* insulin receptor and insulin-like peptides in growth control. Curr Biol. 2001; 11: 213–221. 11250149 10.1016/s0960-9822(01)00068-9

[4] Géminard C, Rulifson EJ, Léopold P. Remote control of Insulin secretion by fat cells in *Drosophila* . Cell Metab. 2009; 10: 199–207. 10.1016/j.cmet.2009.08.002 19723496

[5] Rulifson EJ, Kim SK, Nusse R. Ablation of insulin-producing neurons in flies: growth and diabetic phenotypes. Science 2002; 296: 1118–1120. 12004130 10.1126/science.1070058

[6] Colombani J, Andersen DS, Léopold P. Secreted peptide Dilp8 coordinates *Drosophila* tissue growth with developmental timing. Science 2012; 336: 582–585. 10.1126/science.1216689 22556251

[7] Garelli A, Gontijo AM, Miguela V, Caparros E, Dominguez M. Imaginal discs secrete insulin-like peptide 8 to mediate plasticity of growth and maturation. Science 2012; 336: 579–582. 10.1126/science.1216735 22556250

[8] Fayard E, Tintignac LA, Baudry A, Hemmings BA. Protein kinase B/Akt at a glance. J. Cell Sci. 2005; 118: 5675–5678. 16339964 10.1242/jcs.02724

[9] Gao X, Zhang Y, Arrazola P, Hino O, Kobayashi T, Yeung RS, et al Tsc tumour suppressor proteins antagonize amino-acid-TOR signalling. Nat Cell Biol. 2002; 4: 699–704. 12172555 10.1038/ncb847

[10] Garami A, Zwartkruis FJ, Nobukuni T, Joaquin M, Roccio M, Stocker H, et al Insulin activation of Rheb, a mediator of mTOR/S6K/4E-BP signaling, is inhibited by TSC1 and 2. Mol Cell 2003; 11: 1457–1466. 12820960 10.1016/s1097-2765(03)00220-x

[11] Inoki K, Li Y, Xu T, Guan KL. Rheb GTPase is a direct target of TSC2 GAP activity and regulates mTOR signaling. Genes Dev. 2003; 17: 1829–1834. 12869586 10.1101/gad.1110003 PMC196227

[12] Saucedo LJ, Gao X, Chiarelli DA, Li L, Pan D, Edgar BA. Rheb promotes cell growth as a component of the insulin/TOR signalling network. Nat Cell Biol. 2003; 5: 566–571. 12766776 10.1038/ncb996

[13] Zhang Y, Gao X, Saucedo LJ, Ru B, Edgar BA, Pan D. Rheb is a direct target of the tuberous sclerosis tumour suppressor proteins. Nat Cell Biol. 2003; 5: 578–581. 12771962 10.1038/ncb999

[14] Goberdhan DC, Paricio N, Goodman EC, Mlodzik M, Wilson C. *Drosophila* tumor suppressor PTEN controls cell size and number by antagonizing the Chico/PI3-kinase signaling pathway. Genes Dev. 1999; 13: 3244–3258. 10617573 10.1101/gad.13.24.3244 PMC317204

[15] Hahn K, Miranda M, Francis VA, Vendrell J, Zorzano A, Teleman AA. PP2A regulatory subunit PP2A-B' counteracts S6K phosphorylation. Cell Metab. 2010; 11: 438–444. 10.1016/j.cmet.2010.03.015 20444422

[16] Funakoshi M, Tsuda M, Muramatsu K, Hatsuda H, Morishita S, Aigaki T. A gain of function screen identifies *wdb* and *lkb1* as lifespan-extending genes in *Drosophila* . Biochem Biophys Res Commun. 2011; 405:667–672. 10.1016/j.bbrc.2011.01.090 21281604

[17] Padmanabhan S, Mukhopadhyay A, Narasimhan SD, Tesz G, Czech MP, Tissenbaum HA. A PP2A regulatory subunit regulates *C*. *elegans* insulin/IGF-1 signaling by modulating AKT-1 phosphorylation. Cell 2009; 136: 939–951. 10.1016/j.cell.2009.01.025 19249087 PMC2707143

[18] Vereshchagina N, Ramel MC, Bitoun E, Wilson C. The protein phosphatase PP2A-B' subunit Widerborst is a negative regulator of cytoplasmic activated Akt and lipid metabolism in *Drosophila* . J Cell Sci. 2008; 121: 3383–3392. 10.1242/jcs.035220 18827008

[19] Oldham S, Montagne J, Radimerski T, Thomas G, Hafen E. Genetic and biochemical characterization of dTOR, the *Drosophila* homolog of the target of rapamycin. Genes Dev. 2000; 14: 2689–2694. 11069885 10.1101/gad.845700 PMC317036

[20] Zhang H, Stallock JP, Ng JC, Reinhard C, Neufeld TP. Regulation of cellular growth by the *Drosophila* target of rapamycin dTOR. Genes Dev. 2000; 14: 2712–2724. 11069888 10.1101/gad.835000 PMC317034

[21] Rajan A, Perrimon N. *Drosophila* as a model for interorgan communication: lessons from studies on energy homeostasis. Dev Cell 2011; 21:29–31 10.1016/j.devcel.2011.06.034 21763605 PMC3560414

[22] Wullschleger S, Loewith R, Hall MN. TOR signaling in growth and metabolism. Cell 2006; 24: 471–484. 10.1016/j.cell.2006.01.016 16469695

[23] Hay N, Sonenberg N. Upstream and downstream of mTOR. Genes Dev. 2004; 18: 1926–1945. 15314020 10.1101/gad.1212704

[24] Nagel AC, Fischer P, Szawinski J, La Rosa MK, Preiss A. Cyclin G is involved in meiotic recombination repair in *Drosophila melanogaster* . J Cell Sci. 2012; 125: 5555–5563. 10.1242/jcs.113902 22976300

[25] Nagel AC, Szawinski J, Fischer P, Maier D, Wech I, Preiss A. Dorso-ventral axis formation of the *Drosophila* oocyte requires Cyclin G. Hereditas 2012; 149: 186–196. 10.1111/j.1601-5223.2012.02273.x 23121330

[26] Faradji F, Bloyer S, Dardalhon-Cuménal D, Randsholt NB, Peronnet F. *Drosophila melanogaster* Cyclin G coordinates cell growth and proliferation. Cell Cycle 2011; 10: 805–818. 21311225 10.4161/cc.10.5.14959

[27] Giot L, Bader JS, Brouwer C, Chaudhuri A, Kuang B, Li Y, et al. A protein interaction map of *Drosophila melanogaster* . Science 2003; 302: 1727–1736. 14605208 10.1126/science.1090289

[28] Stanyon CA, Liu G, Mangiola BA, Patel N, Giot L, Kuang B, et al A *Drosophila* protein-interaction map centered on cell-cycle regulators. Genome Biol. 2004; 5(12): R96 15575970 10.1186/gb-2004-5-12-r96 PMC545799

[29] Böhni R, Riesgo-Escovar J, Oldham S, Brogiolo W, Stocker H, Andruss AF, et al Autonomous control of cell and organ size by CHICO, a *Drosophila* homolog of vertebrate IRS1-4. Cell 1999; 97: 865–875. 10399915 10.1016/s0092-8674(00)80799-0

[30] Xu T, Rubin GM. Analysis of genetic mosaics in developing and adult *Drosophila* tissues. Development 1993; 117: 1223–1237. 8404527 10.1242/dev.117.4.1223

[31] Gong WJ, Golic KG. Ends-out, or replacement, gene targeting in *Drosophila* . Proc Natl Acad Sci. USA 2003; 100: 2556–2561. 12589026 10.1073/pnas.0535280100 PMC151379

[32] Reis T, Van Gilst MR, Hariharan IK. A buoyancy-based screen of *Drosophila* larvae for fat storage mutants reveals a role for Sir2 in coupling fat storage to nutrient availability. PLOS Genet. 2010; 6(11): e1001206 10.1371/journal.pgen.1001206 21085633 PMC2978688

[33] Gutierrez E, Wiggins D, Fielding B, Gould AP. Specialized hepatocyte-like cells regulate *Drosophila* lipid metabolism. Nature 2007; 445: 275–280. 17136098 10.1038/nature05382

[34] Grönke S, Mildner A, Fellert S, Tennagels N, Petry S, Müller G, et al Brummer lipase is an evolutionary conserved fat storage regulator in *Drosophila* . Cell Metab. 2005; 1: 323–330. 16054079 10.1016/j.cmet.2005.04.003

[35] Colombani J, Raisin S, Pantalacci S, Radimerski T, Montagne J, Léopold P. A nutrient sensor mechanism controls *Drosophila* growth. Cell 2003; 114: 739–749. 14505573 10.1016/s0092-8674(03)00713-x

[36] Ikeya T, Galic M, Belawat P, Nairz K, Hafen E. Nutrient-dependent expression of insulin-like peptides from neuroendocrine cells in the CNS contributes to growth regulation in *Drosophila* . Curr Biol. 2002; 12: 1293–1300. 12176357 10.1016/s0960-9822(02)01043-6

[37] Park S, Alfa RW, Topper SM, Kim GES, Kockel L, Kim SK. A genetic strategy to measure circulating *Drosophila* insulin reveals genes regulating insulin production and secretion. PLOS Genet. 2014; 10: e1004555 10.1371/journal.pgen.1004555 25101872 PMC4125106

[38] LaFever L, Drummond-Barbosa D. Direct control of germline stem cell division and cyst growth by neural insulin in *Drosophila* . Science 2005; 309: 1071–1073. 16099985 10.1126/science.1111410

[39] Britton JS, Lockwood WK, Li L, Cohen SM, Edgar BA. *Drosophila's* Insulin/PI3-Kinase pathway coordinates cellular metabolism with nutritional conditions. Dev Cell 2002; 2: 239–249. 11832249 10.1016/s1534-5807(02)00117-x

[40] Janssens V, Goris J. Protein phosphatase 2A: a highly regulated family of serine/threonine phosphatases implicated in cell growth and signalling. Biochem J. 2001; 353: 417–439. 11171037 10.1042/0264-6021:3530417 PMC1221586

[41] Bennin DA, Arachchige Don AS, Brake T, McKenzie JL, Rosenbaum H, Ortiz L, et al. Cyclin G2 associates with Protein Phosphatase 2A catalytic and regulatory B’ subunits in active complexes and induces nuclear aberrations and a G1/S phase cell cycle arrest. J Biol Chem. 2002; 277: 27449–27467. 11956189 10.1074/jbc.M111693200

[42] Okamoto K, Li H, Jensen MR, Zhang T, Taya Y, Thorgeirsson SS, et al Cyclin G recruits PP2A to dephosphorylate Mdm2. Mol Cell 2002; 9: 761–771. 11983168 10.1016/s1097-2765(02)00504-x

[43] Hannus M, Feiguin F, Heisenberg CP, Eaton S. Planar cell polarization requires Widerborst, a B' regulatory subunit of protein phosphatase 2A. Development 2002; 129: 3494–3503. 10.1242/dev.129.14.3493 12091318

[44] Wilson C, Vereshchagina N, Reynolds B, Meredith D, Boyd CAR, Goberdhan DCI. Extracellular and subcellular regulation of the PI3K/Akt cassette: new mechanisms for controlling insulin and growth factor signalling. Biochem Soc Trans. 2007; 35: 219–221. 17371242 10.1042/BST0350219 PMC2648506

[45] Vereshchagina N, Wilson C. Cytoplasmic activated protein kinase Akt regulates lipid-droplet accumulation in *Drosophila* nurse cells. Development 2006; 133: 4731–4735. 17079271 10.1242/dev.02659

[46] Salvaing J, Nagel AC, Mouchel-Vielh E, Bloyer S, Maier D, Preiss A, et al The Enhancer of Trithorax and Polycomb Corto interacts with Cyclin G in *Drosophila* . PLOS One 2008; 3(2):e1658 10.1371/journal.pone.0001658 18286205 PMC2243016

[47] Debat V, Bloyer S, Faradji F, Gidaszewski N, Navarro N, Orozco-Terwengel P, et al Developmental stability: a major role for cyclin G in *Drosophila melanogaster* . PLOS Genet. 2011; 7: e1002314 10.1371/journal.pgen.1002314 21998598 PMC3188557

[48] Kurimchak A, Graña X. PP2A counterbalances phosphorylation of pRB and mitotic proteins by multiple CDKs: Potential implications for PP2A disruption in cancer. Genes Cancer 2012; 3: 739–748. 10.1177/1947601912473479 23634261 PMC3636755

[49] Chowdhury D, Keogh MC, Ishii H, Peterson CL, Buratowski S, Lieberman J. Gamma-H2AX dephosphorylation by protein phosphatase 2A facilitates DNA double-strand break repair. Mol Cell 2005; 20: 801–809. 16310392 10.1016/j.molcel.2005.10.003

[50] Kimura SH, Ikawa M, Ito A, Okabe M, Nojima H. Cyclin G1 is involved in G2/M arrest response to DNA damage and in growth control after damage recovery. Oncogene 2001; 20: 3290–3300. 11423978 10.1038/sj.onc.1204270

[51] Zimmermann M, Arachchige-Don AS, Donaldson MS, Dappapiazza RF, Cowen CE, Horne MC. Elevated cyclin G2 expression intersects with DNA damage checkpoint signaling and is required for a potent G2/M checkpoint arrest response to doxorubicin. J Biol Chem 2012; 287: 22838–22853. 10.1074/jbc.M112.376855 22589537 PMC3391138

[52] Kockel L, Kerr KS, Melnick M, Brückner K, Hebrock M, Perrimon N. Dynamic switch of negative feedback regulation in *Drosophila* Akt-TOR signaling. PLOS Genet. 2010; 6:e1000990 10.1371/journal.pgen.1000990 20585550 PMC2887466

[53] Huang J, Manning BD. A complex interplay between Akt, TSC2 and the two mTOR complexes. Biochem Soc Trans. 2009; 37: 217–222. 10.1042/BST0370217 19143635 PMC2778026

[54] Maier D, Marquart J, Thompson-Fontaine A, Beck I, Wurmbach E, Preiss A. In vivo structure-function analysis of *Drosophila* Hairless. Mech Dev. 1997; 67: 97–106. 9347918 10.1016/s0925-4773(97)00117-2

[55] Rubin GM, Spradling AC. Genetic transformation of *Drosophila* with transposable element vectors. Science 1982; 218: 348–353. 6289436 10.1126/science.6289436

[56] Tapon N, Ito N, Dickson BJ, Treisman JE, Hariharan IK. The *Drosophila* Tuberous Sclerosis complex gene homologs restrict cell growth and cell proliferation. Cell 2001; 105: 345–355. 11348591 10.1016/s0092-8674(01)00332-4

[57] Tautz D, Pfeifle C. A non-radioactive *in situ* hybridization method for the localization of specific RNAs in *Drosophila* embryos reveals translational control of the segmentation gene hunchback. Chromosoma 1989; 98: 81–85. 2476281 10.1007/BF00291041

[58] Miron M, Verdú J, Lachance PE, Birnbaum MJ, Lasko PF, Sonenberg N. The translational inhibitor 4E-BP is an effector of PI(3)K/Akt signalling and cell growth in *Drosophila* . Nat Cell Biol. 2001; 3: 596–601. 11389445 10.1038/35078571

[59] Gyuris J, Golemis E, Chertkov H, Brent R. Cdi1, a human G1 and S phase protein phosphatase that associates with Cdk2. Cell 1993; 75: 791–803. 8242750 10.1016/0092-8674(93)90498-f

[60] Hollenberg SM, Sternglanz R, Cheng PF, Weintraub H. Identification of a new family of tissue-specific basic helix-loop-helix proteins with a two-hybrid system. Mol Cell Biol 1995; 15: 3813–3822. 7791788 10.1128/mcb.15.7.3813 PMC230620

