# Supplementary material for: Cyclin G Functions as a Positive Regulator of Growth and Metabolism in Drosophila
Source: PLoS Genet. 2015 Aug 14;11(8):e1005440. doi: 10.1371/journal.pgen.1005440 (PMC4537266; doi:10.1371/journal.pgen.1005440)
Supplement: S1 Fig — (A) Genomic structure of the cycG locus and the neighboring medea and RpL6 genes according to FlyBase (R6.03). Exons are boxed, lighter shades indicate untranslated regions. Only strongly supported transcripts are depicted. The cycG eoC allele was generated by 'ends-out' recombination using the indicated fragments Eo1a and Eo1b (see S1 Text), resulting in the deletion of most of the coding region of cycG. (B) cycG HR7 and cycG eoC mutants behave as protein null by western blot analyses. No CycG protein can be detected in adult heads of neither cycG HR7 nor cycG eoC homozygotes nor cycG HR7 /cycG eoC trans-heterozygotes. In contrast, CycG protein is detected in the heads of wild type (Wt), hs-CycG; cycG HR7 and hs-CycG flies at ambient temperature. Beta-Tubulin served as loading control. (C-C') Size and weight comparison of wild type (Wt) and cycG homozygous (cycG HR7 or cycG eoC) and trans-heterozygous (cycG HR7/cycG eoC) mutant larvae at 126 h of development. Note the smaller size (C) and reduced weight (C') of cycG mutants in comparison to the wild type, whereas no significant differences are seen amongst the cycG mutants. (D-D') Size and weight comparison of wild type (Wt) and cycG homozygous (cycG HR7 or cycG eoC) and trans-heterozygous (cycG HR7/cycG eoC) mutant adult males. Note the smaller size (D) and reduced weight (D') of the cycG mutants in comparison to the wild type. In panels C' and D' error bars denote standard deviation [n = 100 each]. *** p<0.001; ns: not significant according to Student’s T-test. (PDF) [file pgen.1005440.s001.pdf]

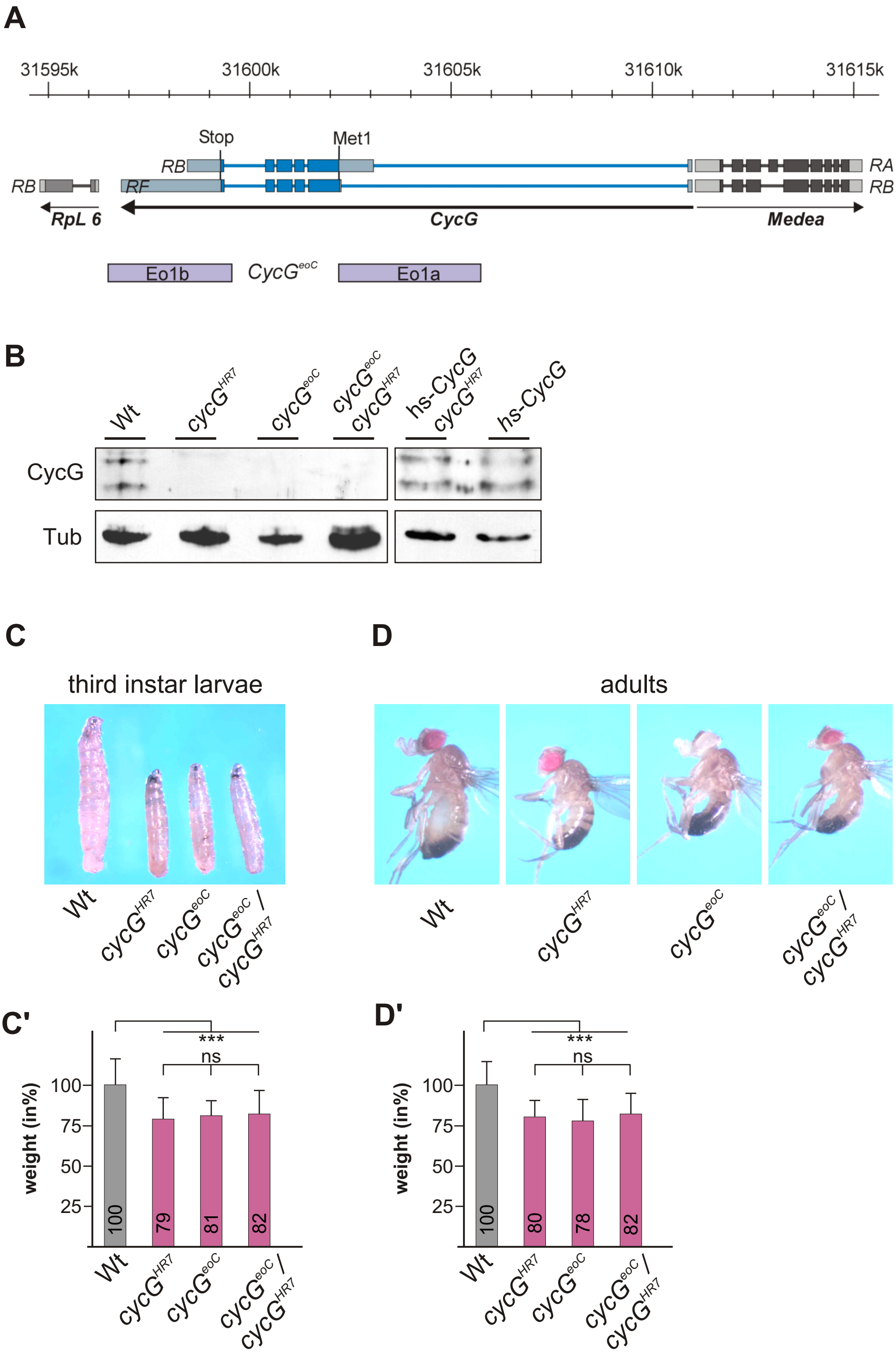
A
31595k
31600k
31605k
31610k
31615k
Stop
Met1
RB
RF
RB
RA
RB
RpL 6
CycG
Medea
Eo1b
CycG^eoC
Eo1a
B
Wt
cycG^HR7
cycG^eoC
cycG^eoC cycG^HR7
hs-CycG cycG^HR7
hs-CycG
CycG
Tub
C
third instar larvae
Wt
cycG^HR7
cycG^eoC
cycG^eoC / cycG^HR7
D
adults
Wt
cycG^HR7
cycG^eoC
cycG^eoC / cycG^HR7
C'
weight (in%)
***
ns
100
75
50
25
100
79
81
82
Wt
cycG^HR7
cycG^eoC
cycG^eoC / cycG^HR7
D'
weight (in%)
***
ns
100
75
50
25
100
80
78
82
Wt
cycG^HR7
cycG^eoC
cycG^eoC / cycG^HR7
