# Supplementary figures and images for: Cyclin G Functions as a Positive Regulator of Growth and Metabolism in Drosophila
Source: PLoS Genet. 2015 Aug 14;11(8):e1005440. doi: 10.1371/journal.pgen.1005440 (PMC4537266; doi:10.1371/journal.pgen.1005440)

**A**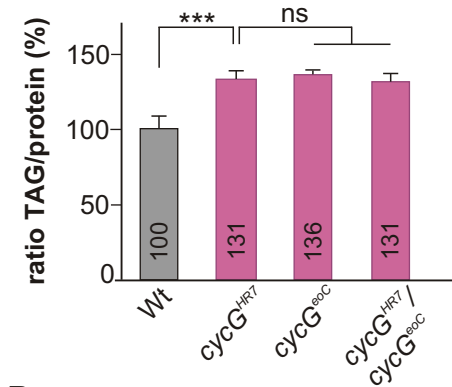**B**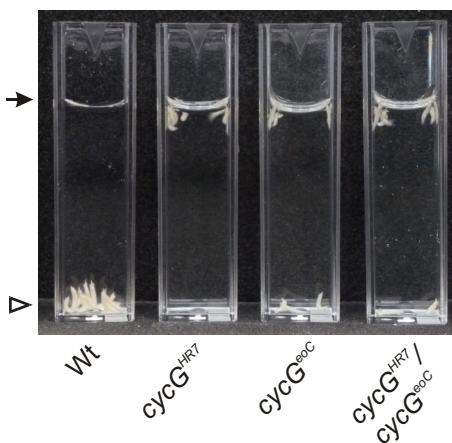**B'**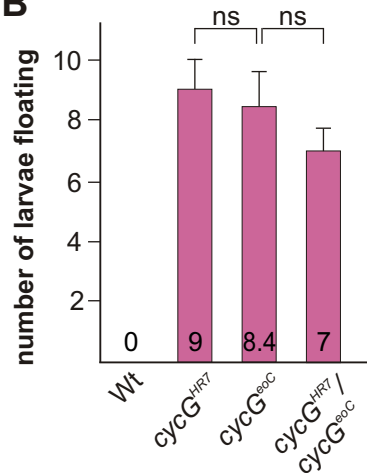**C** Wt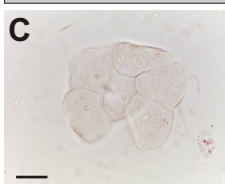**D** *cycG<sup>HR7</sup>*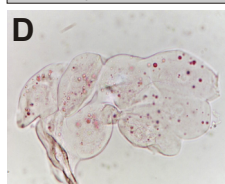**E** *cycG<sup>eoC</sup>*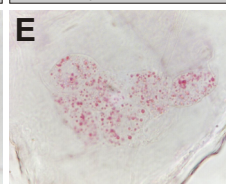**F** *cycG<sup>HR7</sup>/cycG<sup>eoC</sup>*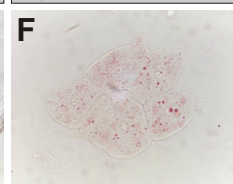

Supplement: S2 Fig — (A) All cycG mutant combinations show an increase of larval TAG content. Histogram depicting TAG content normalized to protein content; wild type (Wt) levels were taken as 100%. In all panels error bars denote standard deviation [n≥3 experiments]. ***p<0.001; ns: not significant according to Student’s T-test. (B-B') Buoyancy test using cycG mutant larvae as indicated. Compared to wild type (Wt), the mutants mostly float (B). Statistical evaluation of the assay repeated five times with ten larvae each (B'). No significant differences were detected between the different mutant combinations according to Student’s T-test; error bars denote standard deviation. (C-F) Comparable lipid droplet accumulation was observed in cycG HR7, cycG eoC and cycG HR7/ cycG eoC mutant larval oenocytes, in contrast to wild type (Wt) control. Scale bar: 20 μm. For a statistical evaluation see S3 Fig. (PDF) [file pgen.1005440.s002.pdf]

% area stained

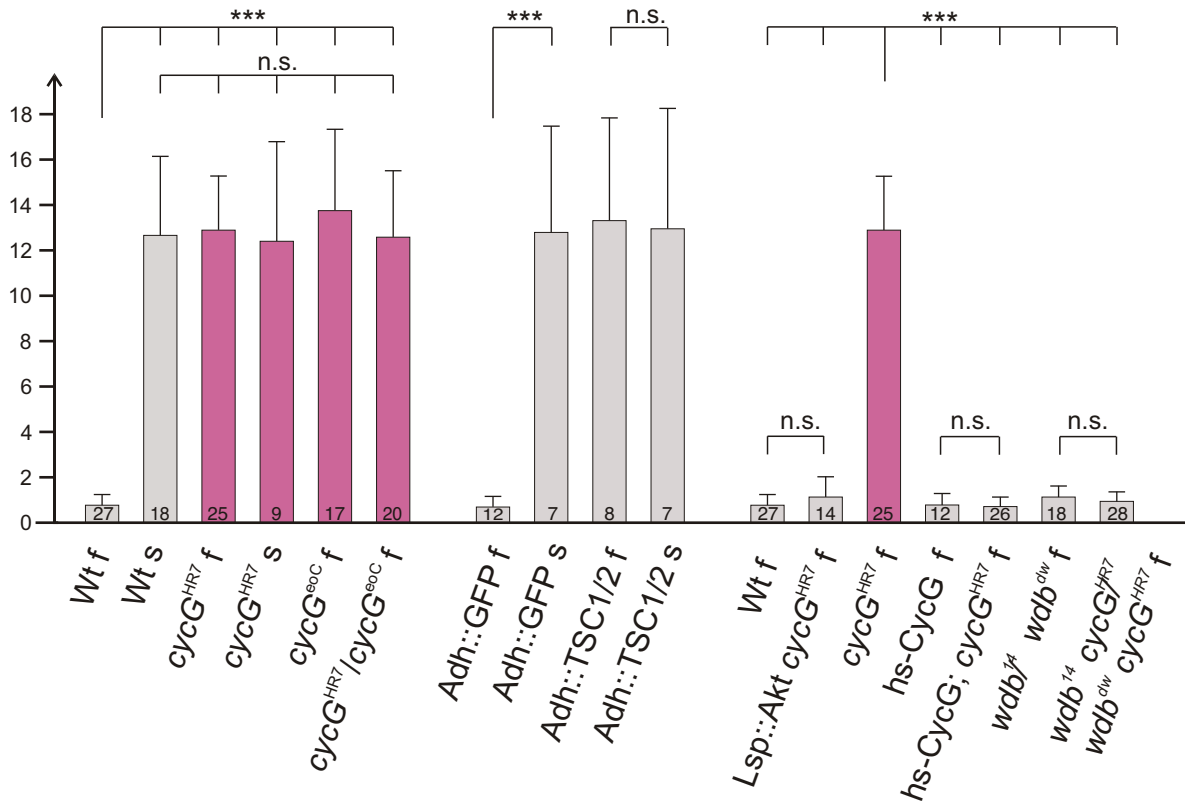

Supplement: S3 Fig — Oenocytes of the given genotypes were analysed (s, starved; f, fed). Staining of oenocytes was quantified as percentage of the stained versus the total area using Image J. Error bars denote standard deviation, the number of cells recorded is indicated. ***p<0.001 and n.s., not significant, according to Student’s T-test. In each instance, there is a highly significant difference between oenocytes that are stained by oil-red-O and those that are not. (PDF) [file pgen.1005440.s003.pdf]

**A**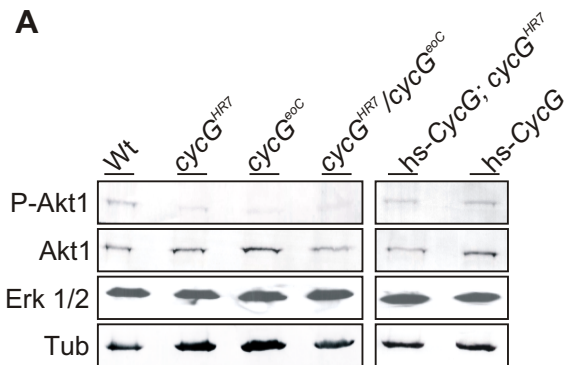**B**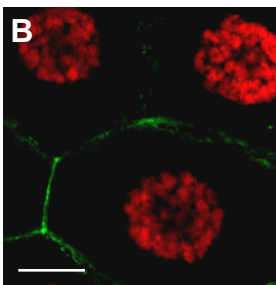**C**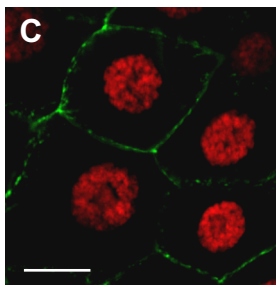**D**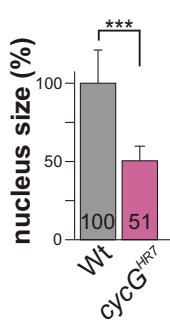**E**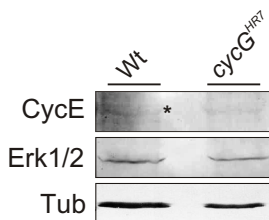**F**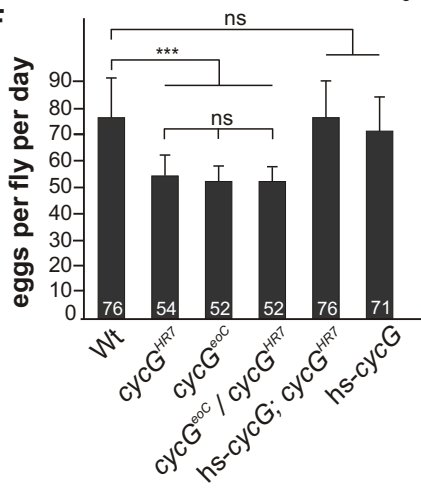

Supplement: S4 Fig — (A) Levels of phosphorylated (P-Akt1) vs. unphosphorylated Akt1 protein were detected in western blots of fly head protein extracts. Compared to the wild type (Wt), cycG mutant animals (cycG HR7; cycG eoC; cycG HR7/cycG eoC) exhibit reduced levels of phosphorylated Akt1 protein. The combination hs-CycG; cycG HR7 shows wild type levels of phosphorylated Akt1 protein at ambient temperature, as does hs-CycG on its own. Erk1/2 and beta-Tubulin (Tub) were used as loading control. (B-C) Salivary gland nuclei were stained with propidium iodide (red); phalloidin staining (green) outlines the cells. Note large polyploid nuclei in the wild type (B). Salivary gland cells of cycG HR7 mutants are smaller, as are the nuclei (C). Scale bar: 20 μm. (D) The nuclear diameter was measured in the central focal section of each nucleus [n = 90]. Wild type control was taken as 100%. Error bars denote standard deviation; ***p<0.001 according to Student’s T-test. (E) Western blot using larval protein extracts of wild type (Wt) and cycG HR7 mutants were probed for CycE protein (*). Erk1/2 and beta-Tubulin (Tub) were used for a loading control. (F) Eggs laid per day in the presence of yeast were counted for wild type (Wt), cycG HR7 or cycG eoC homozygotes, cycG HR7/cycG eoC trans-heterozygotes as well as hs-CycG; cycG HR7 and hs-CycG flies at ambient temperature. 10 females each were aged for two days and mated with 5 wild type males. Eggs were counted every 24 hrs for five consecutive days. The experiment was done in triplicate. cycG mutant females show a significantly reduced egg laying rate compared to wild type, whereas the cycG mutants do not differ among each other. The egg laying defect was rescued in the hs-CycG background at ambient temperature. Error bars denote standard deviation. ***p<0.001, ns: not significant according to Student’s T-test. (PDF) [file pgen.1005440.s004.pdf]

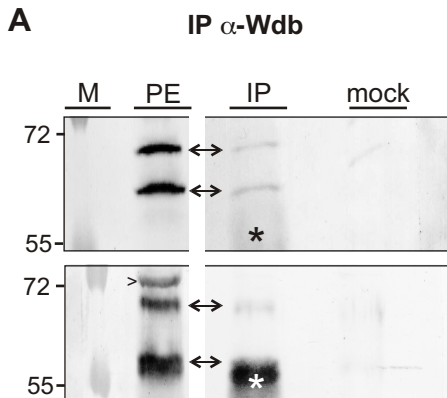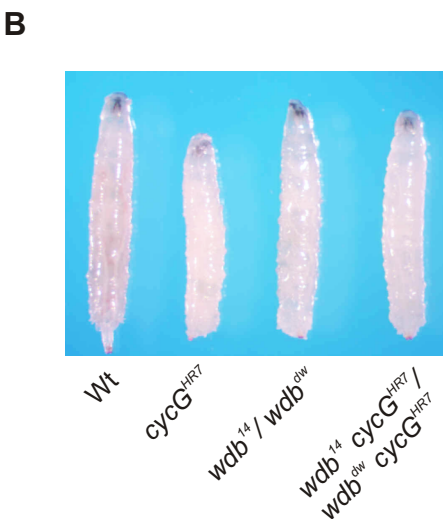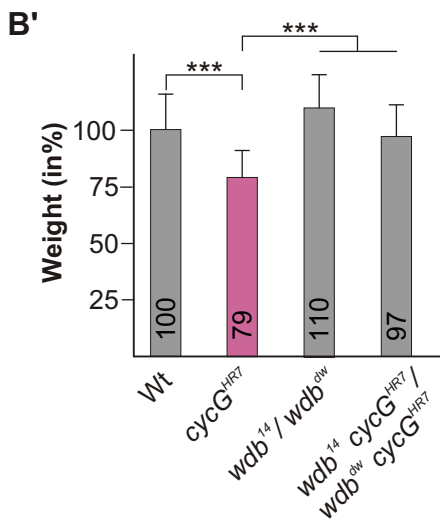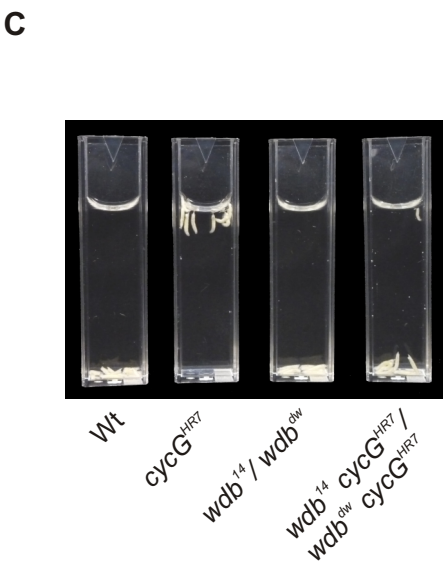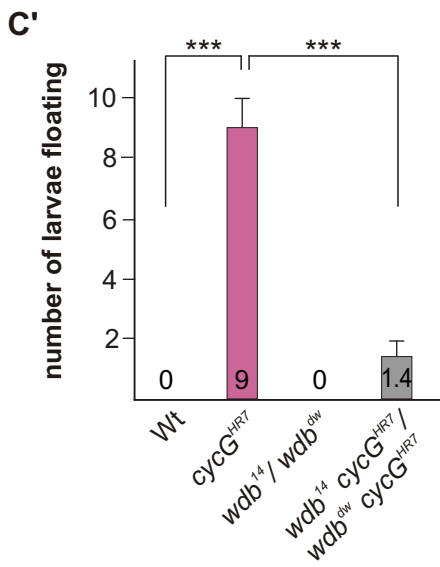

Supplement: S5 Fig — (A) Proteins immunoprecipitated (IP) from embryonic extracts using guinea pig anti-Wdb antibodies were probed for Wdb (anti-Wdb; upper row, arrows) or CycG (anti-CycG; lower row, arrows) using respective rat antisera. The input lane contained 25% of the protein extract (PE) used for the IP. Guinea-pig preimmune serum was used as mock control. The asterisks label unspecific IgG signals. Blots were cut to allow for exposure adjustment of the input. M, size standard in kDa. (B-B') The size and weight deficit of the homozygous cycG HR7mutant is significantly rescued in a wdb mutant background. B) Size comparison of late third instar larvae (126 h after egg deposition). B') The weight of 100 larvae each was measured and is shown relative to the wild type control, which was taken as 100%. Depicted are wild type (Wt), cycG HR7 homozygous mutant, wdb 14 cycG HR7/wdb dw cycG HR7 and wdb 14 / wdb dw animals. Error bars denote standard deviation. ***p<0.001 according to Student’s T-test. (C-C') The number of floating cycG HR7 mutant larvae is strongly reduced in a wdb mutant background (C). (C') Statistical evaluation of the assay repeated five times with 10 larvae each. Error bars denote standard deviation. ***p< 0.001 according to Student’s T-test. (PDF) [file pgen.1005440.s005.pdf]
