# Supplementary material for: Cyclin G Functions as a Positive Regulator of Growth and Metabolism in Drosophila
Source: PLoS Genet. 2015 Aug 14;11(8):e1005440. doi: 10.1371/journal.pgen.1005440 (PMC4537266; doi:10.1371/journal.pgen.1005440)
Supplement: S1 Text — (DOC) [file pgen.1005440.s006.doc]

**S1 Text. Supporting Materials and Methods**

**Generation and verification of the *cycG^eoC^* allele**

To generate a second *cycG* null allele, the 'ends-out' gene replacement method was applied [1], aiming to delete most of the *cycG* coding region. To this end a 3.5 kb genomic fragment from the first intron (Eo1a) and a 3.1 kb genomic fragment from the 3' region of the *cycG* locus (Eo1b) was PCR amplified; they were cloned into the pW25 transformation vector [1]. Primers included restriction enzyme target sites for cloning (bold underlined): 5' fragment: *eoup1a* (***Not*I**): 5'-*AGC A*U***GC GGC CGC*** U*CGA CGT TGT TGG TGC CTA GAT AAA A-3'* and *eolo1a* (***Acc*65I**): 5'- *TAC C*U***GG TAC C***U*GT GAG GGC TGG CCT AGT ACC TGT TG*-3'; 3' fragment (***Asc*I**): *eoup1b*: 5'-*AGC A*U***GG CGC GCC*** U*AAA AGG GGA CTT CAA CTT GAA CTT A*-3' and *eolo1b* (***BsiW*I**): 5'- *TAC C*U***CG TAC G***U*GT ATC GCA ACG TTT TAA ATC TAT TG*-3'. P-element mediated transformation yielded the starting line for homologous recombination that contained the construct on the third chromosome. Homologous recombination, followed by a deletion of the inserted *white* marker gene via Cre/lox recombination, was performed as described before [1]. Four different lines were initially established and tested positive by PCR amplification straddling the deleted region. The *cycG^eoC^* mutant was additionally sequence verified, behaved as a protein null as deduced from western blot analyses and was used in our subsequent analyses.

**Western blot analyses**

To address CycG or CycE protein expression, protein was extracted from 10-15 homozygous wild type or *cycG* mutant larvae and run on a 10% SDS-PAGE followed by Western blotting. For detection guinea-pig anti-CycG antibodies (1:500; [2]), guinea pig anti-CycE antibodies (1:7500, obtained from T. Orr-Weaver; [3]), anti-Erk1/2 antibodies (1:1000) (Cell Signaling Technology; Danvers MA, USA) or mouse anti-beta-Tubulin antibodies (E7; 1:50, DSHB, Iowa, USA) were used.

**Staining of salivary glands**

Salivary glands of staged third instar larvae were stained with propidium iodide as nuclear marker and with FITC-coupled phalloidin (1:100; Sigma Aldrich; Munich, Germany) as cell outline. Tissues were mounted in Vectashield and analyzed with a Zeiss-ApoTome Axio Imager using AxioVision Software for size measurements (Carl Zeiss AG; Oberkochen, Germany).

**Generation of anti-Wdb antibodies**

Guinea pig and rat anti-Wdb antibodies were raised against the whole Wdb protein fused to maltose binding protein (PINEDA; Berlin, Germany). The suitability of the antibodies was tested on imaginal discs overexpressing Wdb and in Western blot analyses of protein extracts from *wdb^14^/wdb^dw^* mutant larvae compared to a wild type control, respectively. Both antibodies were used in a 1:500 dilution.

**Quantification of oenocyte staining**

Image J was used to quantify the stained versus the total area of oenocytes. The total area of each oenocyte was recorded in pixels using the *freehand selection*. The image was inversed, and the *threshold* adjusted to a *brightness* of *100*. In a few instances brightness was reduced (min 80) or increased (max 120), depending on the picture’s contrast. Further settings were *default thresholding method*, *white threshold color, HSB color space* and *dark background*. Stained droplets were framed individually using the *Wand-tool* and their area was recorded in pixels.

**Supporting References**

1. Gong WJ, Golic KG. Ends-out, or replacement, gene targeting in *Drosophila*. Proc Natl Acad Sci USA 2003; 100: 2556-2561.

2. Salvaing J, Nagel AC, Mouchel-Vielh E, Bloyer S, Maier D, Preiss A, et al. The Enhancer of Trithorax and Polycomb Corto interacts with Cyclin G in *Drosophila*. PLOS One 2008; 3(2):e1658.

3. Zhang H, Stallock JP, Ng JC, Reinhard C, Neufeld TP. Regulation of cellular growth by the *Drosophila* target of rapamycin dTOR. Genes Dev. 2000; 14: 2712-2724.
